# Effect of Artificial Selection on Runs of Homozygosity in U.S. Holstein Cattle

**DOI:** 10.1371/journal.pone.0080813

**Published:** 2013-11-14

**Authors:** Eui-Soo Kim, John B. Cole, Heather Huson, George R. Wiggans, Curtis P. Van Tassell, Brian A. Crooker, George Liu, Yang Da, Tad S. Sonstegard

**Affiliations:** 1 Bovine Functional Genomics Laboratory, USDA-ARS, Beltsville, Maryland, United States of America; 2 Animal Improvement Programs Laboratory, USDA-ARS, Beltsville, Maryland, United States of America; 3 Department of Animal Science, University of Minnesota, Saint Paul, Minnesota, United States of America; Auburn University, United States of America

## Abstract

The intensive selection programs for milk made possible by mass artificial insemination increased the similarity among the genomes of North American (NA) Holsteins tremendously since the 1960s. This migration of elite alleles has caused certain regions of the genome to have runs of homozygosity (ROH) occasionally spanning millions of continuous base pairs at a specific locus. In this study, genome signatures of artificial selection in NA Holsteins born between 1953 and 2008 were identified by comparing changes in ROH between three distinct groups under different selective pressure for milk production. The ROH regions were also used to estimate the inbreeding coefficients. The comparisons of genomic autozygosity between groups selected or unselected since 1964 for milk production revealed significant differences with respect to overall ROH frequency and distribution. These results indicate selection has increased overall autozygosity across the genome, whereas the autozygosity in an unselected line has not changed significantly across most of the chromosomes. In addition, ROH distribution was more variable across the genomes of selected animals in comparison to a more even ROH distribution for unselected animals. Further analysis of genome-wide autozygosity changes and the association between traits and haplotypes identified more than 40 genomic regions under selection on several chromosomes (Chr) including Chr 2, 7, 16 and 20. Many of these selection signatures corresponded to quantitative trait loci for milk, fat, and protein yield previously found in contemporary Holsteins.

## Introduction

The availability of relatively high density single-nucleotide polymorphism (SNP) assays allows for the determination of autozygous segments based on runs of consecutive homozygous genotypes. Runs of homozygosity (ROH) would be expected within an individual when both identical haplotypes share a recent common ancestor [1], and this should be correlated to the inbreeding coefficient as defined by the probability that two genes at a locus are identical by descent [2]. A simulation study suggests that the inbreeding coefficient (*F*) estimated from ROH retains variation even in large populations and is likely to be the most powerful method of detecting inbreeding effects from among several alternative estimates of inbreeding [3]. These ideas were supported by McQuillan and colleagues [4] when genomic inbreeding coefficients based on ROH were found to correlate strongly (*r* = 0.86) with pedigree inbreeding coefficients within a specific inbred human cohort. Moreover, an abundance of SNP-based ROH was detected in other ethnic populations explaining the effect of geographical isolation and reduced effective population size on genomic autozygosity [1,5]. The extent of ROH is also affected by genomic regions with decreased recombination activity [6] or high linkage disequilibrium [5]. More recently, ROH methodology has been applied to investigate the influence of genomic autozygosity on survival to old age, which allowed the use of a recessive genetic model to identify effects of rare alleles on disease [7]. Keller and colleagues [8] demonstrated that the odds of schizophrenia increase by 17% for every 1% increase in genome-wide autozygosity supporting a role for multiple recessive or partially recessive alleles in the etiology of schizophrenia.

For ROH investigations in cattle, popular dairy breeds in North America (NA) provide a good model system due to the high frequency of familial relationships within the pedigrees of elite animals. These pedigree complexities commonly result from genetically superior sires being used repeatedly for reproduction by artificial insemination (AI). This type of breeding scheme stimulates genetic improvement for high milk yield including fat and protein content [9], and has resulted in about a two-fold increase in productive traits for a typical modern cow relative to its predecessor from approximately 10 generations ago. The cost of this mating strategy for NA Holstein and Jersey cattle included increases in potentially deleterious genomic autozygosity known as inbreeding depression [10]. In principle, inbreeding does not change allele frequency in a random mating population without mutation, migration, or selection [11]. However, the intense artificial selection in NA dairy breeds appears to have altered allele frequency patterns by increasing identical by descent (IBD) haplotypes. Therefore, heavily used elite sires could possibly have influenced the distribution of ROH with effects comparable to genetic drift; a process that can cause large changes in allele frequency over a short time period [12]. In a previous study, ROH sampling from a set of elite Jersey cattle demonstrated existence of a preferential distribution of ROH [13]. There also was a high correlation between ROH and autozygosity (*r* ~ 0.7), which agreed with the results discussed above for studies of ROH in human populations [4].

The purpose of the current study was to elucidate the effect of selection, inbreeding, and genetic drift on autozygosity in NA Holsteins during the last 40 years. The comparison of SNP-based ROH was between a contemporary group of commercial pedigree animals, an experimental line of cows selected for milk production, and a control line of animals mated to avoid inbreeding but unselected for milk production since 1964 [14,15]. It was expected that the genomic autozygosity in this latter group would be significantly lower than animals under selection, and thus present a unique opportunity to characterize ROH status and distribution due to breed development prior to the intense selection for milk that took place in the latter half of the 20^th^ century. This within breed comparison of genome autozygosity differs considerably from previous reports using a more contemporary sampling of elite commercial Holstein sires outside of NA to find selection signatures [16,17]. In addition, the two groups under selection in this study were produced in different environments, but had substantial overlap in ancestry due to common AI sires used to boost milk production. The unselected group was not influenced by these sires. The comparison of ROH patterns from selected groups could also possibly provide insight into the effect of selection on the genome over varying periods of time and determine if the direction of selection was similar. Finally, the relationship between high ROH regions and economic traits under artificial selection is assessed between each group.

## Materials and Methods

### Animals and genotypes

The study’s resource population was made up of the following three groups: 1) the majority of the 150 founding sires the University of Minnesota control line started in 1964 and some of the unselected cows descended from this line (Group I; N = 299); 2) contemporary cows selected by Holstein USA from elite herds in Wisconsin, Iowa, Vermont, Pennsylvania, Virginia and Florida (Group II-A; N = 1,634); and 3) some of the more contemporary cows descended from the University of Minnesota selection line for milk production started in 1964 (Group II-B; N = 151) [14]. The entire pedigree of the resource population included about 40,000 animals, and phenotypes for most animals (>80%) were available from the National Dairy Database (USDA, ARS, Animal Improvement Programs Laboratory). Illumina’s BovineSNP50 bead chip assay (San Diego, CA) was used to generate the marker scores from the 41,951 polymorphic SNP with minor allele frequency (MAF) greater than 1% analyzed in this study. Some of this genotypic data has been used previously to complete a genome wide association analysis in Holsteins [18], and all of the genotypes have been used previously to calculate genome predictions of genetic merit (http://www.aipl.arsusda.gov/). Only SNP located on autosomal chromosomes were selected for autozygosity determination, and SNP positions were based on bovine genome coordinates from the UMD 3.1 genome sequence assembly model (http://www.cbcb.umd.edu/research/bos_taurus_assembly.shtml). 

### Runs of homozygosity and genomic inbreeding coefficient estimation

An intact homozygous genomic region or run of homozygosity (ROH) was defined by a state of contiguous homozygous genotypes that was equivalent to or more than 50 or 100 consecutive SNP depending on the threshold used for detection. The primary hypothesis is that ROH in the selected groups (Group II-A and II-B) would higher have levels of autozygosity than unselected group (Group I) in the same genomic locations. Acceptance of this hypothesis could support the conclusion that artificial selection or increased inbreeding affects ROH patterns in Holsteins. Group means were calculated from the total length of ROH per individual. The statistical tests for differences between groups were determined using ROH information that included all animals and ROH positions. 

The inbreeding coefficient (*F*_*P*_) of an animal [19] was calculated using pedigrees. The genomic inbreeding coefficient (*F*_*G*_) was estimated by the sum of ROH length of an individual divided by the total length of the autosomes [3]. Briefly, the genomic inbreeding coefficient assumed two haplotypes of each homozygous region (>50 or 100 SNPs) were IBD regardless of pedigree relationships between an individual and its ancestors. The detection of continuous haplotype regions and comparison of homologous genomic regions between individual and ancestors were done using Perl scripts. The similarity of genomic region between two individuals was measured based on the sum of mismatches between an individual and its ancestors: 


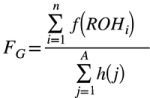


where *f* (*ROH_i_* ) = size of *i* th runs of homozygosity *ROH*_*i*_ *, n* = total number of intact homozygous genomic regions of an individual genome, *h*(*j*) = genome size of *j* ^th^ Chr, *A* = number of bovine autosomal chromosome (N = 29).

The correlation of *F*_*G*_ with *F*_*P*_ was calculated using either all animals or excluding 100 founder animals for which *F*_*P*_ was artificially set to 0. The inbreeding coefficients of founder animals were also approximated from the intercept of linear regression between genomic and pedigree inbreeding coefficients excluding founder animals. 

To summarize the level of local autozygosity in population, the locus autozygosity was defined based on ROH status: 


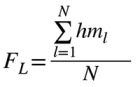


where *N* is total individual number and *hm*_*l*_ is locus autozygous status (0 or 1) of *l* th individual. The mean differences of locus autozygosity were compared to evaluate the overall autozygosity level among three groups. Autosomal autozygosity was also assessed using *F*_*L*_ to survey the effect of selection and inbreeding on autozygosity for each chromosome. Again, 100 animals in Group I were excluded for calculating the correlation coefficient because *F*_*P*_ of these animals had been set to zero in the absence of pedigree information.

### Comparisons of genomic location of F_L_ between groups

The proportions of each group with ROH measuring less than 50 (>0.3 Mb) and 100 (2 Mb) SNP were compared to examine ROH characteristics, and eventually a 50 SNP threshold was used for locus autozygosity. The number of ROH was log-transformed because the data were not normally distributed in all groups. The locus autozygosity (*F*_*L*_) was compared for each locus based on the ROH status of the SNP locus (0 or 1) to test the genetic effect. Then *F*_*L*_ in the same locus between three groups was compared using a chi-square test. The statistical test of *F*_*L*_ for each locus between two groups was also presented across the genome. Although some individuals in the sample have long ROHs (>50 Mb) that can be indicative of very recent common ancestry, all animals were kept in the dataset for further comparisons. A comparison using the chi-square test considers only differences of *F*_*L*_ at a locus between groups regardless of the direction of the autozygosity change or distribution of *F*_*L*_ in the whole genome, and a statistical threshold was calculated by genome-wide permutation test [20]. Key characteristics used to define the presence of ROH islands included regions showing high *F*_*L*_ level (e.g. *F*_*L*_ > 0.2) and high (s.d.) of *F*_*L*_. 

We propose a comparative approach based on a standardized transformation of the extended haplotype homozygosity suggested by Voight et al. [21]. To compare autozygosity, the log ratio of *F*_*L*_ values at each locus *L* was compared between two groups: ln(*FR*_*L*_) = ln(*F*_*L, Group1*_ / *F*_*L, Group2*_). In contemporary Holsteins, the intensive use of a few elite animals has increased similarity of haplotypes across the breed, causing high haplotype homozygosity at specific genomic regions. Therefore, the extreme value of ln(*FR*_*L*_) reflects the change of allele frequency at locus *L* with respect to haplotype homozygosity. This ratio summarizes homozygosity attributable to recent inbreeding or selection at a locus (*F*_*L*_), and the resulting value is standardized as:

$$s\ln(FR_{L})=\frac{\ln(FR_{L})-median(\ln(FR_{L}))}{stdev(\ln(FR_{L}))}$$

where stdev(ln(*FR_L_*)) is the standard deviation of the locus ratio (ln(*FR_L_*)). The median was used to standardize the ratio to minimize the effect of extreme ratios of locus autozygosity [22].

### Change of locus autozygosity

The change of locus autozygosity (*ΔF*_*L*_) was modeled using a logistic regression. Analysis of the model was performed using the ROH of locus to detect significant autozygosity changes as a means to assess the trend in ROH during last 50 years. The logistic regression model was:

$$hm=\frac{e^{\alpha +\beta by}}{1+e^{\alpha +\beta by}}$$

where *hm =* autozygous status of locus (0, 1), *by* = birth year of an animal, α = the intercept of the equation, and β = the change of annual autozygosity. 

The autozygous status of a locus included both homozygous SNP genotypes. Statistical thresholds for a genome-wide search of autozygosity and genotype frequency change were determined empirically using a permutation test [20]. The experiment-wise critical values (1% and 5%) were obtained by 1,000 permutations, and analysis was performed separately for each group. 

### Genetic effect of frequent haplotypes on traits

Haplotypes were used to examine associations between the most frequent allele and milk yield traits. Haplotypes were defined using a 50 SNP sliding window, and phase was decided using fastPHASE [23]. The genetic effect of the most frequent haplotype (*Q*) against the other haplotype (q) on trait was evaluated across the genome. Use of a sliding window haplotype instead of ROH-based methods in this analysis allowed an evaluation based on additive effect of the most frequent haplotype. The correlation (r) between ROH and homozygosity of the most frequent haplotype was 0.85. Associations between haplotypes and traits were evaluated using a model without polygenic effect following that method reported by Cole and colleagues [18]: 

y=μ+Xb+e

where *y* is the PTA-based breeding value for milk, fat or protein yield; μ is the overall phenotypic mean; and *e* is the residual. The incidence matrix X indicates the number of the most frequent haplotype (0, 1 or 2) in an individual. The size of vector *b* was variable for the haplotype-based model (size of *b* = *N* × 1) where N is the number of haplotypes included in the model (*N* = 3). In this model, the effects of the haplotypes except the most frequent one were set to 0, and the genetic effect of the most frequent haplotype was estimated. This analysis is independent of selection statistically. In addition, the association test of the most frequent haplotype and birth year was performed using a model suggested for analyzing changes in homozygosity. Distribution of birth year in each group is shown in Figure S1. In the logistic regression above, homozygous status defined by ROH was replaced with the number of the most-frequent haplotype (0, 1, or 2). The threshold for genome-wide significance was determined by a 1000-fold permutation test [20].

### Signature of selection using extended haplotype homozygosity (iHS)

The evidence for positive selection was determined by calculating the value of the standardized integrated extended haplotype homozygosity (iHS) for each marker [21]. This test measures the relative decay of extended haplotype homozygosity of the ancestral and derived core alleles. The ancestral type alleles were obtained from a previous study [24]. The absolute value of iHS was calculated at 39,791 test loci with a maximum bracket size of 5 Mb and standardized within group. The large absolute value of iHS (>3) reflects long haplotypes subjected to strong recent selection for the core allele. This analysis was conducted within each of the 3 groups.

## Results

### Runs of Homozygosity

To survey the effects of selection and random drift on whole-genome autozygosity, ROH were detected using two different SNP threshold parameters (50 and 100 SNP windows) across 2,033 genomes representing three different selection groups of Holsteins: 1) Group I (control or unselected since 1964), 2) Group II-A (contemporary Holsteins from 1975-2008), and 3) Group II-B (1964 selection line for milk production). The detection statistics for ROH number and size are found in Table 1. A total of 85,031 ROH were detected at a 50 SNP threshold, and the mean length of homozygous fragments was 6.61 Mb. At 100 SNP, the total number detected decreased to about 39,000 ROH, while the mean length increased to about 10 Mb. The most notable difference between parameters was the order of magnitude increase in minimum ROH size at a 100 SNP threshold leading to a significant decrease in detecting ROH smaller than 5 Mb (Table 1). ROH less than 5 Mb accounted for approximately 53% of all fragments detected at a 50 SNP threshold, but contributed less than 30% of the cumulative ROH length (Figure S2). Identical ROH were found when autozygosity length exceeded 10 Mb regardless of the SNP threshold parameter. Based on these observations, a 50 SNP threshold appeared better for autozygosity detection and defining ROH potentially derived from older common ancestors. 

**Table 1:** ROH Detection and Size Statistics^**1**^.

	**Group I**		**Group II-A**		**Group II-B**		**Total**	
Threshold^2^	**50**	**100**	**50**	**100**	**50**	**100**	**50**	**100**
ROH	7808	3392	71063	32865	6160	2911	85031	39168
Mean^3^	31.1^*^	13.5	43.5	20.1	40.4	19.5	41.8	19.3
s.d.^4^	19.2	12.6	22.2	13.5	21.0	11.9	22.1	13.4
Mean	6.26^**^	10.08	6.65	10.33	6.67	10.20	6.61	10.29
Median	4.37	7.97	4.72	8.19	4.82	8.17	4.69	8.17
s.d.^5^	5.45	5.68	5.68	6.60	5.53	6.34	5.65	6.57
Minimum	0.22	2.71	0.21	2.05	0.21	2.39	0.21	2.05
Maximum	64.2	64.22	87.13	87.13	55.04	55.04	87.13	87.13

1 ROH size results corresponding to last five rows in the table are in Mb

2 SNP window size used to define ROH threshold - 50 or100 SNP

3 Mean number of ROH per individual

4 s.d. of ROH number per individual animal

5 s.d. = standard deviation of ROH length

^*^ (*p* < 0.0001); ^**^ (*p* < 0.00001) based on t-tests

On an individual animal basis, the average genome-wide ROH count was 41.8 with a range between 2 and 194 ROH per animal across groups (Table 1). The maximum size of ROH was 87.13 Mb found on Chromosome (Chr) 8 in a contemporary Holstein cow (Group II-A). The mean number of ROH (50 SNP) per individual in Group I (31.11±19.2) was significantly different (t-test, *p* < 0.0001) from Groups II-A (43.5±22.2) and II-B (40.4±21). There was also a significant difference (*p* < 0.00001) in mean ROH length when comparing Group I to Group II-A or II-B, and some of this difference could be attributed to the increased frequency of ROH fragments of 2-3 Mb detected in Group I and 4-6 and 10-20 Mb in the selected groups (Figure S3). No obvious difference was found between the two selected groups relative to ROH length (*p* ~ 0.2). 

### Inbreeding coefficients and ancestral relationships

Pedigree inbreeding coefficients (*F*_*p*_) ranged from 0 to 0.299 across the three groups (Table S1). The mean *F*_*p*_ was 0.041 under the assumption of no genetic relationship among founder animals (*F*_*p*_ = 0). The ratio of founder animals, defined by *F*_*p*_ = 0, were 0.32, 0.06 and 0.01 in Groups I, II-A and II-B, respectively. In order to quantify the difference in inbreeding level among the three groups, *F*_*p*_ was compared using nonparametric statistical methods. *F*_*p*_ was significantly lower (*p* < 0.001) in Group I (0.016) than Groups II-A (*F*_*p*_ = 0.045) and II-B (*F*_*p*_ = 0.043) under selection, and no significant difference was detected between Groups II-A and II-B (*p* > 0.1). These results were consistent with expectations considering the correlations in common ancestors between groups. Comparison of common ancestors suggested a high genetic correlation (*r* = 0.95) between Groups II-A and II-B (Figure S4A) with an approximate 80% overlap for the 100 most-used sires between groups. In contrast, Group I originated from unique ancestors with little or no relation to each other or the ancestors of Groups II-A and II-B based on the limits of our pedigree analysis (Figure S4B). 

The mean genomic inbreeding coefficients (*F*_*G*_) were higher than *F*_*P*_ across all groups when calculated under the assumption of no genetic relationship among founder animals (Table S1). Comparing the differences between *F*_*G*_ of the selected and unselected groups revealed *F*_*G*_ was significantly higher (*p* < 0.0001) in Group II animals. The correlations between *F*_*G*_ and *F*_*P*_ ranged from 0.59 to 0.68 (Table S2), and were slightly stronger at a 50 SNP threshold. Removing 100 founders with an assumed *F*_*P*_ = 0 from the analysis had little effect on the correlation. 

In attempt to determine a more accurate level of inbreeding for our Holstein groups at the onset of selection, animals with an *F*_*P*_ artificially set to zero were excluded from a linear regression analysis between *F*_*G*_ and *F*_*P*_ to infer approximate inbreeding coefficients for the founding animals. The range of slope was 0.85 to 1.05, and the intercept was between 0.02 and 0.04 (Table S3), which reflects the more probable level of inbreeding for founding animals born 10-20 generations ago in all three groups.

### Locus autozygosity

**Figure 1:**
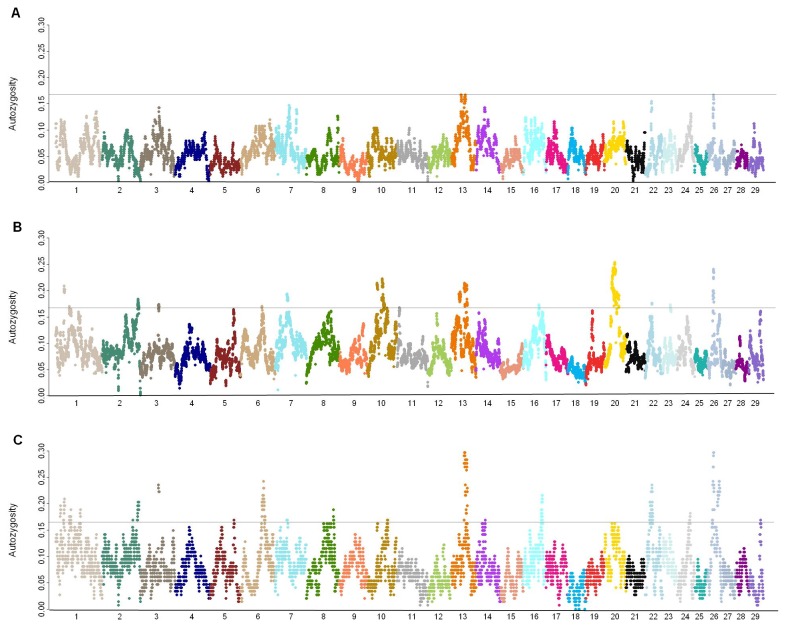
Genome-wide patterns of *F*_*L*_ within Groups. Manhattan plots of autozygosity were generated using the *F*_*L*_ values (y-axis) for each SNP locus relative to its genome coordinate (x-axis; Chr indicated) for A) Group I, B) Group II-A, and C) Group II-B. The grey horizintal line indicates the threshold for *F_L_* > 0.16, which represents approximately the top 2.5% of autozygosity detected.

Locus autozygosity (*F*_*L*_) and mean *F*_*L*_ per chromosome were determined in an attempt to evaluate autozygosity of genomic regions due to inbreeding and/or selection (Table S4). Examination of *F*_*L*_ revealed that Groups II-A and II-B have a higher general level of autozygosity compared to unselected animals of Group I. As expected, *F*_*L*_ was not uniformly distributed across any chromosome (Figure 1), and was much more variable among the selected groups with the s.d. of *F*_*L*_ ranging from 0.02 to 0.08. On a genome-wide basis, correlations between *F*_*L*_ locations revealed the two selected groups shared common patterns of locus autozygosity (*r* = 0.67) in contrast to patterns seen in Group I (*r* = 0.33 to 0.34). While mean *F*_*L*_ never exceeded 0.09 in Group I, there clearly were some conserved patterns of increased *F*_*L*_ shared by all groups on Chr 13 and 26 (Table S5 and Figure 1). There were 13 regions on 11 chromosomes identified to have higher autozygosity only in the selected groups with similar conserved patterns of regional autozygosity reaching the top 3% of all *F*_*L*_. Maximum *F*_*L*_ (0.297) was located between 47.4-48.1 Mb on Chr 13 in Group II-B, and between 36.5-36.6 Mb on Chr 20 in Group II-A (*F*_*L*_ = 0.254). Only the largest value of *F*_*L*_ in Group I (0.167) on Chr 26 was within the top 2% of all *F*_*L*_ values across groups.

To investigate differences in *F*_*L*_ between groups further, we applied a standardized log ratio to *F*_*L*_ (Figure S5). For example, a ratio of Group II-A to I yielding a positive value for sln(FR) indicates higher *F*_*L*_ in Group II-A, whereas a negative value represents relatively higher autozygosity in Group I, which was presumably not subject to intensive artificial selection. The absolute values of sln(FR) above 3.0 are reported in Table S6. The maximum and minimum sln(FR) values between Group II-A and I were 5.39 and -2.87, respectively, indicating a maximum range of positive selection (positive value) and probably the maximum effect of genetic drift (negative value). In these group comparisons, a total 403 sln(FR) values exceeded 3.0, while there were no sln(FR) values smaller than -3.0. Meanwhile, the comparison between the selection and control lines from the Minnesota Herd (Group II-B and I) identified several regions representing extreme negative sln(FR) values < -3.0 on Chr 11, 18, 24, 26, and 29 (Table S6). When the two selected groups were compared, more than 10 regions were found to have positive values of sln(*FR*) > 3.0 in Group II-A with no directional selection of this magnitude detected in Group II-B. 

**Figure 2:**
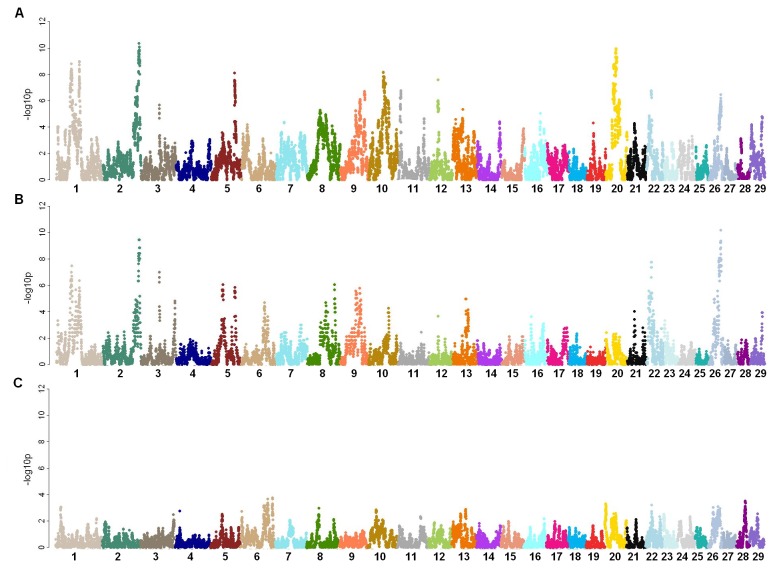
Two way comparisons of *F*_*L*_ between Groups. Manhattan plots of comparative autozygosity were generated using *–log*_*10*_ *p* of chi-square tests between two groups (y-axis) for each SNP locus relative to its genome coordinate (x-axis; Chr indicated) for A) Group II-A versus I, B) Group II-B versus I, and C) Group II-A versus II-B.

To further assess the effect of selection on ROH, a chi-square test of *F*_*L*_ was applied to account for the differences between groups (Figure 2). Among more than 30 regions that appeared under selection in each group compared to Group I (Table 2), we found 13 consensus regions in Groups II-A and II-B (-*log*_*10*_ *p* > 4). No substantial differences in *F*_*L*_ between Groups II-A and II-B were detected (Figure 2C), which is not surprising because these groups share nearly the same set of influential sires. In comparison to the analysis of *F*_*L*_ ratios between groups, the chi-square results agreed with eight regions under significant selection on Chr 1, 2, 9, 21, and 22 found in Group II-A and B relative to Group I (Table S6).

**Table 2:** Difference of ROH (*F*_*L*_) between selected and unselected groups.

	**Group II-A *vs* I**			**Group II-B *vs* I**		
**Chr**	**Candidate region^1^**	**Max (Mb)**	**Difference of *F*_*L*_ (p-value)^2^**	**Candidate region^1^**	**Max (Mb)**	**Difference of *F*_*L*_ (p-value)^2^**
1	2.85-4.05	3.64	0.09 (4.05)	-	-	-
	47.30-67.73	52.25	0.15 (8.81)	47.70-67.33	53.53	0.17 (7.49)
	76.51-84.94	79.59	0.14 (8.98)	74.20-82.03	79.59	0.14 (8.98)
2	111.58-134.31	131.46	0.17 (10.34)	111.58-134.31	131.46	
3	69.90-70.39	70.26	0.12 (5.66)	69.90-71.23	70.26	0.12 (5.66)
	-	-	-	116.98-117.15	117.15	0.10 (4.79)
5	-	-	-	42.68-46.74	45.11	0.14 (6.05)
	96.58-104.48	97.32	0.14 (8.09)	97.22-101.40	98.83	0.14 (5.84)
6	19.58-20.51	20.51	0.09 (4.18)	-	-	-
	-	-	-	81.55-83.15	82.31	0.16 (4.67)
8	40.59-57.56	49.04	0.09 (5.07)	-	-	-
	61.51-67.96	64.12	0.10 (4.91)	63.95-65.30	64.11	0.12 (4.69)
	87.07-88.81	87.07	0.10 (4.09)	-	-	-
	-	-	-	94.41-97.45	94.41	0.15 (6.07)
9	57.54-62.15	59.31	0.09 (4.73)	57.54-64.54	60.47	0.12 (5.41)
	69.14-79.99	72.21	0.08 (5.19)	71.11-79.99	79.99	0.12 (4.91)
10	36.11-38.75	37.66	0.11 (4.52)	-	-	-
	48.42-65.47	55.32	0.15 (7.83)	-	-	-
	70.59-74.06	70.59	0.12 (6.01)	70.48-71.99	71.10	0.13 (4.25)
11	5.01-7.51	6.92	0.13 (6.76)	-	-	-
	88.26-88.90	88.83	0.09 (4.64)	-	-	-
12	33.6-37.01	35.12	0.13 (7.59)	-	-	-
13	11.83-12.19	12.19	0.09 (4.57)	-	-	-
	20.62-21.61	20.62	0.09 (3.83)	-	-	-
	30.26-33.46	32.62	0.10 (4.15)	-	-	-
	36.65-39.02	36.73	-0.09 (4.67)	-	-	-
	-	-	-	47.55-47.92	47.55	0.18 (4.97)
	-	-	-	55.75-56.09	55.75	0.16 (4.13)
14	77.27-77.93	77.75	0.09 (4.41)	-	-	-
16	58.60-60.37	60.37	0.11 (5.04)	-	-	-
	71.43-71.77	71.55	0.09 (71.43)	-	-	-
18	-	-	-	39.25-41.18	40.00	-0.07 (9.83)
20	20.49-49.96	46.41	0.13 (6.09)	-	-	-
21	26.94-27.93	27.22	0.07 (4.28)	26.94-27.30	27.14	0.08 (4.01)
22	7.39-10.56	9.27	0.09 (4.87)	6.99-18.25	15.97	0.17 (7.76)
	13.99-18.25	14.95	0.12 (6.76)	-	-	-
	-	-	-	23.55-23.92	22.50	0.15 (4.09)
26	-	-	-	19.51-20.1	19.91	0.16 (4.95)
	35.26-43.15	41.85	0.12 (6.47)	30.96-43.15	41.68	0.22 (10.19)
29	41.33-43.01	41.71	0.10 (4.27)	43.43-43.84	43.65	0.12 (3.93)

1 Gap smaller than 1 Mb between two regions was regarded as continuous region

2 Chi-square test

### Change of locus autozygosity (*Δ*F_L_)

**Figure 3:**
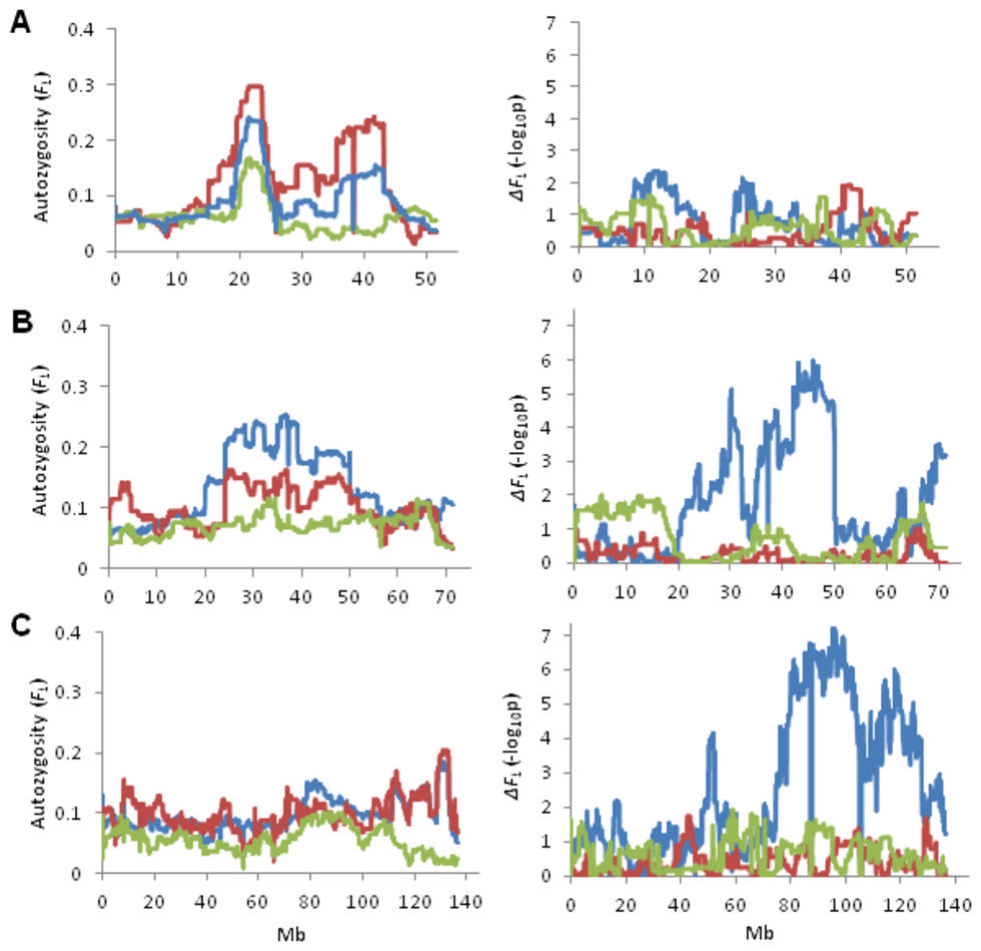
Chromosome plots comparing *F*_*L*_ and *ΔF*_*L*_ across groups. *F*_*L*_ and *ΔF*_*L*_ statistics (y-axis) were plotted against Chr coordinates on A) Chr 26, B) 20, and C) 2. Green, blue, and red lines represent Group I, II-A and II-B, respectively.

To evaluate the effects of selection on genomic diversity, we compared the autozygosity of selected and unselected populations using a generalized linear model. This analysis should reveal regions under recent selection relative to any trends of increasing *F*_*L*_. The association of homozygous status and birth year (*ΔF*_*L*_) was plotted for each group (Figure S6). Of 28 regions showing high autozygosity, significant changes of autozygosity (*p* < 0.01) since the 1960s were found in 12 regions for Group II-A (Figure S6B); whereas weak (Figure S6C) and no evidence (Figure S6A) of changing autozygosity were found in Groups II-B and I, respectively. For example, in the region between 20-25 Mb on Chr 26, *ΔF*_*L*_ was constant in high ROH regions across groups, reflecting consistent selection that maintained autozygosity (Figure 3A). On Chr 20, a long extended haplotype of homozygosity (*F*_*L*_ > 0.16) was found in the region between 35-50 Mb in Group II-A (Figure 3B). Notably, the *F*_*L*_ has increased constantly during the last 50 years, which was supported by association test of homozygous status and birth year. The largest difference of homozygosity between selected and the unselected groups was found at 130 Mb on Chr 2, where the most significant change of *F*_*L*_ was detected in the region between 80-110 Mb in Group II-A (Figure 3C). This suggests that recent selection and inbreeding in Group II-A were more likely to affect homozygosity for the region encompassing 80-130 Mb on Chr 2. 

To gain further insight on changes caused by recent selection, the genome-wide, *ΔF*_*L*_ in Group II-A was plotted with comparisons of *F*_*L*_ between selected (Group II-A) and unselected (Group-I) groups (Figure S7). In addition to the cases described above, there were considerable differences detected between group statistics on Chr 1, 5, 7, 9, 10, 16, and 29. Most regions showing substantial discrepancies were found based on comparison of ROH between selected and unselected populations. However, the regions on Chr 7 and 16 were detected only based on significant *ΔF*_*L*_. 

### Effect of selected haplotype on economic traits

**Figure 4:**
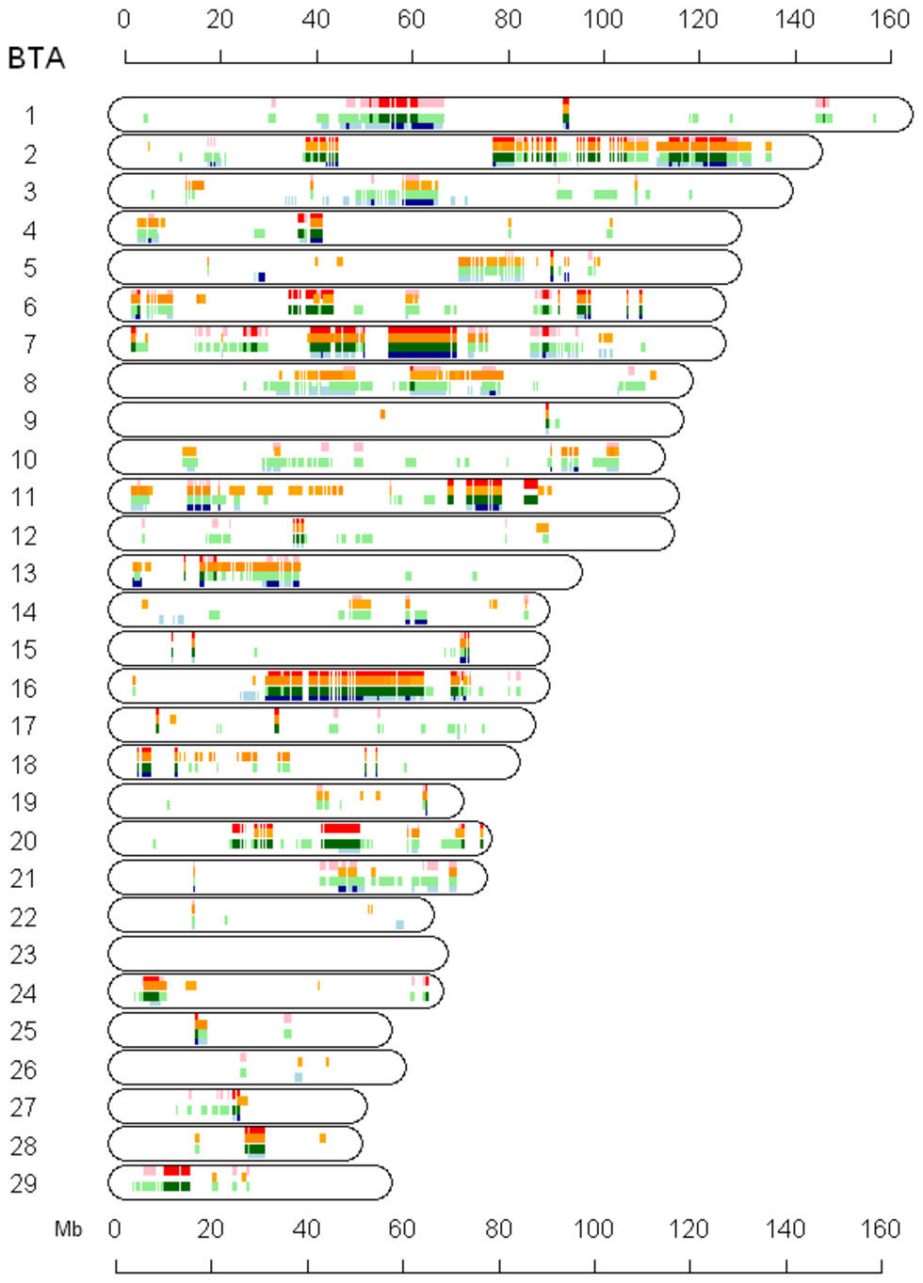
Associations between the most frequent haplotype and birth year and milk yield traits. Associations are plotted against chromosomal regions. Red, blue, orange, and green bars denote an association between Group II-A haplotypes and birth year, milk yield, fat, and protein, respectively (light color = suggestive at p<0.05, dark color = significant at p<0.01).

Although ROH is based on the sum of various homozygous haplotypes, the most frequent haplotype accounts for the majority of sub-chromosomal autozygosity. The animals used in this study have been selected for higher milk production, implying correlations between economic traits and ROH that are influenced by selection. However, additive genetic effects of homozygous haplotypes (or ROH) on traits cannot be directly estimated. Therefore, prior to this association test, we investigated the relationship between major haplotypes and ROH. The highest correlation (*r* ~ 0.7) was found between autozygosity of the most frequent haplotype in each 50 SNP sliding window and *F*_*L*_ that was obtained from the sum of autozygosity based on ROH. Specifically, the most frequent haplotype found within an ROH island is expected to be associated with production, because selection for increased milk, fat and protein yields has been the primary focus of genetic improvement. The additive genetic effect of the most frequent haplotype was evaluated across the genome in Groups II-A relative to production traits under the assumption that all alleles have additive effects of 0 except for the most frequent haplotype (Figure 4). Likewise, the regions showing the most significant changes of autozygosity (-*log*_*10*_ *p* > 4.3) were compared with the associations between most frequent haplotypes and traits in Group II-A and B (Table 3). In summary, several regions (Chr 1, 2, 7, 11, 16, 20) with significant *ΔF*_*L*_ overlapped with the associations between a most frequent haplotype and a yield trait in Group II-A. As noted before, *F*_*L*_ has increased in the region between 80 and 130 Mb on Chr 2 during the last 40 years in Groups II-A and II-B. The most common haplotype (50 SNP window) had a frequency greater than 0.35, which correlated with affects on milk, fat, and protein yield in Group II-A. Moreover, in many cases the region representing apparent differences in *F*_*L*_ between selected and unselected groups were overlapped with those genomic regions explaining variation in traits (Figure S8). In contrast, analyses of Groups II-B (Figure S9) and I (Figure S10) suggested weak evidence of association between the most frequent haplotype or ROH and milk yield traits. 

**Table 3:** Comparison between *ΔF*_*L*_ and the most frequent haplotype-trait association in Group II-A.

**Chr**	**Pos (Mb)**	***ΔF*_*L*_^1^**	***F_L_***	**Association^2^**	**Trait^2^**	**Coding region^3^**	**Candidate genes^4^**
1	29.808	4.298	0.204	-	-	*GBE1*	*-*
	32.739	4.269	0.202	-	-	*CADM2*	*-*
	49.533	4.717	0.159	5.9	Milk	*ALCAM*	*MYH15*
	64.032	4.03	0.110	**10.43**	Protein	*IGSF11*	*B4GALT4*
2	51.035	4.049	0.090	4.28	Milk	*SNRPD1*	*-*
	51.895	4.171	0.088	-	-	*ZEB2*	*GTDC1*
	109.106	4.156	0.108	**8.09**	Fat	*FN 1*	*IGFBP2*
	126.957	4.053	0.094	**14.29**	Fat	*ARID1A*	*E2F2, FGR*
7	57.334	4.146	0.133	**24.72**	Fat	*POU4F3*	*RBM27*
	60.257	5.011	0.121	**25.27**	Fat	*HTR4*	*PPP2R2B*
	62.934	5.113	0.110	**17.87**	Fat	*ANXA6*	*ADRB2*
	65.047	4.537	0.103	**18.42**	Fat	*GLRA1*	*GPX3*
8	46.155	4.033	0.105	**8.61**	Protein	*PTAR1*	*PTAR1*
	61.915	4.024	0.137	**10.45**	Fat	*GRHPR*	*GRHPR*
9	87.889	4.093	0.124	**14.95**	Protein	*PPIL4*	*-*
	88.112	4.181	0.125	5.03	Protein	*LRP11*	*-*
10	97.708	4.374	0.125	5.92	Protein	*FLRT2*	*-*
	99.331	4.307	0.136	**6.26**	Protein	*MIR2293*	*PIDA6*
11	77.826	4.033	0.081	**9.41**	Milk	*APOB*	*GDF7*
	79.052	4.021	0.080	5.97	Fat	*TTC32*	*OSR1*
12	33.603	5.017	0.151	5.71	Fat	*ATP8A2*	
	39.152	4.72	0.106	-	-	*PCDH9*	*MAK1*
13	26.47	4.65	0.149	**17.94**	Fat	*FZD8*	*MYO3A*
	34.981	4.461	0.188	**20.38**	Fat	*SVIL*	*-*
14	28.625	4.423	0.118	-	-	*ASPH*	*NKAIN3*
	33.999	4.265	0.094	-	-	*PRDM14*	*PREX2*
	34.764	4.056	0.080	5.57	Fat	*LACTB2*	*-*
16	57.715	8.156	0.159	**13.39**	Protein	*RABGAP1L*	*RABGAP1L*
	60.366	9.179	0.166	**13.40**	Protein	*CACNA1E*	*-*
20	29.899	4.899	0.231	**7.69**	Fat	*PARP8*	*MRPS30*
	30.216	5.093	0.234	**7.24**	Protein	*CCDC115*	*FGF10, GHR*
	38.675	4.291	0.240	-	-	*SPEF2*	*PLRL*
	49.811	4.404	0.171	5.17	Milk	*ZAR1L*	*CDH 6,9,10*
21	67.458	4.201	0.076	5.03	Fat	*RTL1*	*-*
	67.675	4.041	0.076	**13.54**	Fat	*Multiple MIRs*	*Multiple MIRs*
22	15.89	4.073	0.150	4.29	Protein	*MIR2368*	*VIPR1*
	17.578	4.667	0.143	4.76	Protein	*SETD5*	*OXTR*

1 Adjusted P=0.01 is equivalent to - log_10_p = 4.6.

2 These columns represent associations between haplotypes and traits at loci with significant *ΔF*_*L*_. Adjusted P = 0.05 (- log_10_p ~ 6) and P = 0.01 (- log_10_p ~ 9) are highlighted in bold. Only - log_10_p > 4 is shown.

3 Annotated gene located nearest to the candidate loci

4 Positional and functional candidate genes within the candidate region that are suggested by annotation tool (DAVID, http:// http://david.abcc.ncifcrf.gov/). Gene participating in metabolism, signaling, or cell cycle is shown.

### Signatures of selection: Standardized iHS

To understand the timing of selection further, we performed the iHS test separately for Groups I, II-A, and II-B. The extended haplotype homozygosity analysis suggested that 1,655 loci have been selected (|iHS| > 2) on every chromosome in Group II-A (Figure S11B). A region was defined as recently selected when multiple signals (>10) with |iHS| > 2 were located within 0.3 Mb of each rather than depending on a single high value of |iHS| (>3). Approximately half of the top 2% of high |iHS| windows overlapped with regions under selection identified by analysis of haplotype homozygosity change and trait-haplotype association test. While Group II-A and II-B were both selected for higher milk yields for decades, iHS analysis found few common SNPs under selection in both groups (Figure S12). However, iHS identified several consensus regions influenced by selection, including a signature of selection at 130 Mb on Chr 2 that spans ~5Mb in Group II-A and II-B. In addition for the two selected groups, consensus signatures of selection were found on Chr 7 (93 Mb), 10 (49 Mb), 20 (25 Mb), and 24 (33 Mb). 

Unexpectedly, we obtained more loci with standardized |iHS| > 2 in Group I (1,865) than in Group II-A or II-B, where high |iHS| indicate broad regions (>10 to 20 Mb) on Chr 3, 7, 13, 17, and 22 (Figures S11 and S13); which were partially shared by regions with |iHS| > 3 in Group II-A. In particular, |iHS| analysis suggests that the broad region encompassing 22-70 Mb of Chr 13 was under selection in Group I. Furthermore, we discovered over 200 consensus |iHS| > 2 in Group I and II-A by direct comparison of scores for each locus across the genome, most of which were located on Chr 7, 10, 13, and 20 (Table S7).

## Discussion

Many popular livestock breeds have been established within the past four centuries; experiencing genetic events such as consanguinity, population bottlenecks, and moderate selection before modern animal breeding programs started during the 20^th^ century. Each of these types of genetic events can greatly change genome autozygosity for individuals in a specific population. In this study, we hypothesized that industry standards for Holstein mating systems used since the 1960s would result in unique ROH patterns between elite commercial (Group II-A) and experimental animals under differing selection for milk production (Groups I and II-B). Current industry standards adjust predicted transmitting abilities to reflect the effects of expected future inbreeding [25] by penalizing genetic merit for animals that are highly related to the population while crediting outcross animals. Even though these practices reduce inbreeding levels [26], inbreeding is inevitable for efficient artificial selection on additive traits to improve production ability. Thus, a high proportion of elite modern Holsteins (Group II-A) should have consensus ROH due to common ancestors within 5 or 6 generations carrying superior alleles at specific genome locations, even though the mean inbreeding level is typically not over 0.1 [27]. Our findings on ROH frequency and size distribution between Holstein groups support this hypothesis, and demonstrate ROH analysis is a viable method for assessing genomic variation in livestock. 

First, the mean ROH number per animal across groups (~40) was similar to that found in consanguineous human populations [4,6], but the distribution of ROH sizes in humans (1-5 Mb) was much smaller than those in NA Holsteins (mean of about 6 Mb). This observation could be the result of breed formation bottlenecks coupled with constant selection and unavoidable inbreeding. Group I had significantly fewer ROH per average genome, and the mean ROH size was significantly smaller, even though this difference was only 0.4 Mbp per ROH on average (Table 1). Failure to detect ROH of 2-5 Mb across groups using a 100 SNP threshold was probably due to most 100 marker intervals being larger than 5 Mb. Furthermore, the lower SNP window size (50 SNP) allowed us to account for an additional 30% to the total ROH size and infer that some of the smaller ROH regions have grown as levels of inbreeding have increased over the past few generations. Interestingly, the trends in Group I ROH patterns provide a “snapshot” of genomic autozygosity in NA Holsteins just prior to the intensive use of selection based on genetic merit, because these animals (Group I) were kept in a mating system based only on inbreeding avoidance since the 1960s. No significant differences in frequency could be detected by ROH size category between groups (Figure S3) suggesting selection pressure for production caused the increase in ROH size for Group II-A rather than random drift caused by inbreeding. This phenomenon is further supported by ROH size increases in specific locations affecting milk production (Figure 4). 

Overall, the ROH statistics suggest that across the breed population about 10% of the Holstein genome is autozygous. However, not every ROH is attributable to identical by descent (IBD) haplotypes, because some identical haplotypes are probably originated from unrelated ancestors based on pedigree information. The correlation (*r* < 0.7) between ROH (*F*_*G*_) and pedigree-based inbreeding (*F*_*P*_) coefficients between our Holstein groups supports this possibility. Likewise, VanRaden and colleagues [28] found a correlation of only 0.59 between pedigree and genomic inbreeding for Holsteins born since 1990. Our correlations also corresponded well with those reported in other cattle breeds, but were lower than those found in consanguineous human populations (*r* = 0.8 to 0.9). For example, inbreeding coefficients based on ROH (<1-2 Mb) were considerably higher than pedigree based inbreeding coefficients in a previous cattle study [29]. Another recent ROH survey in U.S. Jersey cattle found a correlation between *F*_*G*_ and *F*_*P*_ of about 0.7 [13]. Our findings were also comparable to a correlation (*r* = 0.8) between pedigree [30] and genomic inbreeding coefficients (Kim et al., unpublished data) obtained in a closed Angus herd, where most animals in this herd (n = 600) originated from approximately 20 founders. In all of these cases, *F*_*G*_ was greater than pedigree-based inbreeding coefficient (*F*_*P*_). A practical explanation for these findings is that the inbreeding levels for founding animals of these populations were not *F*_*P*_ = 0, and the genomic inbreeding coefficient probably includes undocumented inbreeding in the time period between breed formation and pedigree recording. Removing animals with *F*_*P*_ = 0 in our study did not significantly improve the correlation *F*_*G*_ and *F*_*P*_. A regression to calculate true inbreeding found levels equivalent to 0.02 and 0.04 in the founding animals, and incorporation of this information into future analyses could improve the correlation between *F*_*G*_ and *F*_*P*_. 

Previous ROH studies by human geneticists suggested differences in terms of the cumulative ROH length, and a positive correlation between ROH number and the population-specific level of consanguinity [6]. In our study, comparisons of locus autozygosity (*F*_*L*_) patterns between groups are in good agreement with population history. Because few animals in Group I share common ancestors with Groups II-A and II-B, it was not surprising to observe unique patterns of autozygosity in Group I with *F*_*L*_ patterns in Group II-B being similar to that found in Group II-A. Therefore, differences in *F*_*L*_ patterns between selected and unselected populations support the expectation that selection of superior animals affects the formation of ROH. Selection of animals in Groups II-A and II-B since the 1960s has produced tremendous phenotypic changes and reshaped the landscape of ROH in several regions of the genome. Differences between these two selected groups were not revealed by *F*_*L*_ comparison using chi-square analysis. Although changes were small between Groups II-A and B, sln(FR) analysis resulted in the detection of 10 potential regions under selection after the 1980s. As expected, no significant changes in ROH due to selection were detected in Group I, and also in Group II-B. The lack of apparent change in ROH within Group II-B in contrast to the change identified in Group II-A may be explained as a consequence of the shorter sampling period of animals (Group II-B: 1987 to 2003 versus Group II-A: 1975 to 2007). The small population size (n = 150 versus n = 299) may also have provided lower statistical power to detect selection using logistic regression. 

The amount of improvement in milk production depends on changes in frequency of selected alleles, as well as the presence of favorable alleles in founder animals. Using logistic regression analysis, large changes of *F*_*L*_ level (-log_10_p > 4) were detected in more than 10 genomic regions, some of which were not found in comparisons between groups. While the largest value of *F*_*L*_ was not high (0.2) on Chr 7 compared to the other regions, autozygosity has increased consistently since selection programs were started. 

Signatures of selection have been identified using many different methods in diverse cattle breeds [15,31–34], whereas, only a few studies focused on selection occurring during the last few decades in cattle [15,17]. The high-ROH genomic regions in NA Holsteins were consistent with signatures of selection detected by EHH with high core haplotype frequency (>0.25) in German Holsteins [16]. Approximately two-thirds of regions under selection in German Holstein overlapped with high-ROH regions defined as *F*_*L*_ > 0.16 in U.S. Holsteins (Group II-A and II-B). Consensus across long extended haplotypes was also found between U.S. and Israeli Holsteins [16]. The agreement of some signatures of selection within an international breed is expected considering the periodic flow of Holstein germplasm from NA during the 20th century. Specifically, the obvious evidence of recent selection on Chr 20 is concordant in all Holstein studies. In contrast, consensus selection signatures were found between 80 and 90 Mb on Chr 6 in German and Israeli cattle, while neither high levels of ROH (*F*_*L*_) nor the change of autozygosity (*ΔF*_*L*_) were found in the corresponding region in our study. In U.S. Holstein cattle, a moderately frequent (frequency~0.3) long haplotype was found in this region with no notable changes since the 1960s. The detailed objective of selection and environment within these various studies may differ, resulting in different patterns of the shared haplotype segment.

As shown in our study, several ROH regions showing relatively high levels in contemporary cattle were maintained since the 1960s, suggesting that selection increased haplotype frequency during the early 20^th^ century or even further back in time. For example, the high ROH region found on Chr 26 appeared in all groups, including the unselected group. Nowadays, advances in reproductive technology have enabled world-wide germplasm exchanges in a short time period. Therefore, those consensus selected genomic regions discovered in two separate continents are not unusual considering the low effective population size of Holsteins [35].

Identifying evidence of recent positive selection in domesticated animals provided information on genome response to strong directional selection from domestication and artificial selection [31]. When long-term selection was examined, the period of inbreeding caused neutrality test statistics such as Fay & Wu’s H statistics [36] to deviate from expectations under neutrality, mimicking the effect of selection [32]. Changes of autozygosity due to genetic drift are not easily distinguishable from high autozygosity due to selection. Thus, we assume that differences in ROH patterns based on direct comparison between two groups under selection may still potentially reflect drift. The observed ROH pattern among the selected groups appears to be nearly identical, which suggests that the selection of influential sires born between the 1960s and 1980s plays a crucial role in detection of the changes of autozygosity or the most common haplotypes. An alternative comparison method proposed in our study, standardized log ratio (sln(FR)), allowed the detection of probable genetic drift in the unselected population. When selected Holstein groups were compared with the unselected group, extreme sln(FR) scores in the selected groups were substantially greater than the values obtained in the unselected group. Even though genetic drift affected ROH, the effect of drift was restricted across the genome. However, the role of genetic drift was not negligible when a small population (Group II-B) was compared to the unselected group. The extreme value of sln(FR) between Group II-B and I ranged from approximately -4 to +4, implying genetic drift due to the small population size of Group II-B. Understanding the selective forces on a population could be achieved better by comparative study between genome wide associations and signatures of selection [37]. This approach would support evidence that some ROH islands emerged by selection rather than random drift. During the last few decades, the genetic selection of U.S. Holstein cattle for the improvement has had adverse effects on fertility as measured by daughter pregnancy rate (DPR) [38], which is probably related to ROH. However, the relationship between local autozygosity and DPR has not clarified in cattle. 

An association between yield traits and alleles under selection is inevitable due to the tremendous increase in milk yield resulting from the artificial selection program used for decades in commercial dairy cattle production. For haplotype-trait association tests, only the most frequent haplotype was assumed to have an additive effect and contribute to high ROH in groups under selection. The 50 SNP sliding window resulted in ~30 haplotypes, whereas only the most frequent haplotypes (frequency > 0.1) account for most of the observed haplotype homozygosity. Restricting every haplotype but one to have an additive effect of 0 is an unrealistic assumption for the analysis of long-term selection effects in a natural population. However, it is a reasonable assumption that the most frequent haplotypes in high ROH regions affect milk production when considering that most increasing or high ROH were discovered in commercial U.S. Holsteins. Indeed, in selected Holsteins a frequent haplotype contributing to ROH changes is associated with genetic improvement of yield traits. When comparing genome-wide association (GWA) test results conducted using a population almost identical to Group II-A [18], the significant changes of autozygosity showed a similar pattern with the GWA for milk yield, particularly the regions associated with considerable change of ROH on Chr 1, 2, 7, and 20. However, the current analysis appears to produce potential false positives when it is applied to low-ROH regions, and to decrease the power of association testing when multiple alleles influencing the same trait are under selection. The methodology used in this analysis can identify only QTL under recent artificial selection. Andersson and Georges [39] reported that selective sweeps assisted in the identification of most QTL identified in previous livestock studies. 

The extended haplotype homozygosity test, iHS, measures the decay of identity of haplotypes that carry a designated core allele, allowing inference of selected alleles with higher frequency than expected relative to their haplotype length [40]. The iHS method has been successfully applied to discover selected alleles at intermediate frequency (0.7 to 0.9), while almost-fixed alleles (>0.95) are difficult to detect using the standardized iHS approach [22]. The changes in haplotype frequency since 1960s were investigated using individual information including birth year of animals, which enables monitoring of changes in genomic homozygosity. Almost no iHS score higher than 3 were detected when selected alleles were at a relatively high frequency (>0.9).

In selected groups, |iHS| score appears to be consistent with the region representing substantial changes in homozygosity during the last 5 decades. Unexpectedly, we found a large number of SNPs with |iHS| score > 2 in the unselected group, providing evidence of selection before artificial insemination and modern breeding programs were initiated. This observation may be reasonable because iHS analysis is independent of the time of occurrence of selection. In Group I, haplotype homozygosity has not changed significantly during the last 40 years, which is consistent with the history of Group I being unselected for higher milk, fat, or protein yield. Genetic drift also may provide an alternative explanation of the result considering the long range of signals spanning 10 to 20 Mb regions in relatively small populations and the influence of autozygosity originating from recent common ancestors that were not under artificial selection. 

A favorable variant of the *DGAT1* gene was subjected to selection from the 1960s to the 1980s, with little or slightly backward selection after the 1980s in the U.S. Holstein cattle [15], providing an explanation of the weak evidence of selection for *DGAT1* in this study. Despite the strong evidence of recent selection in some regions of the genome identified in this study, most regions are not narrow enough to propose one or even a few candidate gene(s) under selection. However, some regions included genes reported in previous studies, for example, the oxytocin receptor gene (*OXTR*) on Chr 22 which is well-known for its role in milk secretion from the mammary gland [41]. A previous genome-wide scan explaining variation in milk production identified a region at 120 cM on Chr 2 [42] that corresponds to a 90-120 Mb region under recent selection in the current study. The annotation tool DAVID (http://david.abcc.ncifcrf.gov/) was used to identify several candidate genes located in this region. For example, fatty acid binding protein 3 (*FABP3*) gene located at 122 Mb of Chr 2 is one of the most abundant isoforms in bovine mammary tissue, and its expression is dependent on the stage of lactation [43]. On Chr 20, growth hormone receptor (GHR, 31 Mb) and prolactin receptor (*PRLR*, 39 Mb) genes known to be associated with milk production and growth, are found in the large region under strong positive selection in Group II-A. More than 20 other genes located between 43 and 48 Mb may have been influenced by selection during the last 50 years, but not all genes in this region are fully annotated. 

The analysis used in this study can exploit a limited number of historical recombination events; therefore, an additional approach will be necessary to narrow candidate regions. During the last several decades, the genetic ability of dairy cattle has improved tremendously by selection, but the corresponding changes in allele frequencies and genomic homozygosity in response to this selection have not been clarified. The selection signatures can be detected using direct comparison between a few number of elite sires and their offspring [44], while our study assumes inheritance from multiple influential founders in the contemporary Holsteins. 

The selection signatures may provide an optimal selection tool minimize loss of alleles important for milk yield in Holstein cattle, while selecting for new functional traits. The results of this study suggest that ROH produced by recent artificial selection is a useful signature of selection in U.S. Holsteins, which is supported by associations between milk yield traits and haplotypes under selection. However, it is also possible that the comparison of ROH between selected and unselected groups may confound selection and founder effects because selected and unselected groups shared only a few consensus ancestors. The small number of founders can affect the overall autozygosity of the current Holstein population due to the intensive use of AI, particularly in populations under selection. If ancestral animals used for AI shared similar haplotypes since inception of modern genetic improvement programs, a relatively large change in ROH level would not be expected. Another scenario of emerging ROH is that selection has increased the frequency of less common haplotypes which originated from only a few founders. 

In conclusion, our study demonstrated the existence of ROH due to breed formation on Chr 13, 20, and 26. Selection for milk production has continued to extend ROH on Chr 20, while also increasing ROH on other chromosomes of importance that include discrete regions on Chr 1, 2, 7, and 16. Recently, the primary objective of selection in Holsteins has been changing from higher yield to other economic traits such as net merit, productive life, and fertility using genomic predictions [45], which will change genomic features of ROH even further in the future.

## Supporting Information

Table S1 **Summary statistics of *F*_*P*_ and *F*_G_.**
(DOCX)

Table S2 **Correlations (r) between *F*_*P*_ and *F*_G_.**
(DOCX)

Table S3 **Linear regression of *F*_*G*_ on *F*_P_ .**
(DOCX)

Table S4 **Mean locus homozygosity (*F*_*L*_).**
(DOCX)

Table S5 **Genomic regions reaching top 3% of all *F*_*L*_ across groups.**
(DOCX)

Table S6 Genomic intervals representing differences in *F*_*L*_ between groups^*^.
(DOCX)

Table S7 **Summary of genome-wide |iHS|.**
(DOCX)

Figure S1 **Distribution of Birth Year across groups.** The total animal counts (y-axis) is plotted against birthyear (x-axis) for each group.
(TIF)

Figure S2 **Distribution of ROH size across groups.** The total counts of ROH detected across groups with ROH sizes binned on the x-axis and counts indicated on the y-axis using a 50 (red) or 100 (blue) SNP threshold.
(TIF)

Figure S3 **Frequency of ROH sizes detected by group.** ROH lengths were binned by size within group by threshold of a A) 50 SNP or B) 100 SNP window. The y-axis represents frequency of ROH length detected as a percentage of all ROH detected within a group. Length bins are grouped on the x-axis.
(TIF)

Figure S4 **Correlation of common pedigree ancestors between groups.** The appearance of genotyped descendants from common influential sires were counted and expressed as a percentage of the entire group. The graphs comparing the percentage influence from common sires includes: A) common sires from Groups II-A (y-axis) versus II-B (x-axis) and B) Group II (y-axis) versus Group I (x-axis).
(TIF)

Figure S5 **Genome wide plot of comparative *F*_L_.** Manhattan plots were generated using the standardized log ratio of *F*_*L*_ values (y-axis) for each SNP locus relative to its genome coordinate (x-axis; Chr indicated) for A) Group II-A vs I, B) Group II-B vs I, and C) Group II-A vs II-B.
(TIF)

Figure S6 **Genome-wide changes of autozygosity within Groups.** Manhattan plots of change in autozygosity were generated using (*ΔF_L_*) for each SNP locus (y-axis) relative to its genome coordinate (x-axis; Chr indicated) for A) Group I (1953~2006), B) Group II-A (1975~2007), and C) Group II-B (1987~2003).
(TIF)

Figure S7 **Comparison of change of autozygosity and differential selection of autozygosity.** Genome-wide plots by chromosome of*–log*_*10*_ *p* values (y-axis) of the change of autozygosity (*ΔF*_*L*_ - blue dots) of Group II-A and differential selection on autozygosity (orange bars) between Groups II-A and I.
(TIF)

Figure S8 **Comparison between change of the most frequent haplotype and haplotype-trait associations in** Group II-A. Genome-wide Manhattan plots by chromosome of*–log*_*10*_ *p* values (y-axis) of the association between the most frequent haaplotype and haplotype trait associations. Plots are for the following traits A) Milk, B) Fat, and C) Protein, while D) plots haplotype by birth year.
(TIF)

Figure S9 **Comparison between change of the most frequent haplotype and haplotype-trait associations in** Group II-B. Genome-wide Manhattan plots by chromosome of*–log*_*10*_ *p* values (y-axis) of the association between the most frequent haaplotype and haplotype trait associations. Plots are for the following traits A) Milk, B) Fat, and C) Protein, while D) plots haplotype by birth year.
(TIF)

Figure S10 **Comparison between change of the most frequent haplotype and haplotype-trait associations in Group I.** Genome-wide Manhattan plots by chromosome of*–log*_*10*_ *p* values (y-axis) of the association between the most frequent haaplotype and haplotype trait associations. Plots are for the following traits A) Milk, B) Fat, and C) Protein, while D) plots haplotype by birth year.
(TIF)

Figure S11 **Genome-wide integrated extended haplotype homozygosity.** Absolute values of iHS were plotted across the genome. The standardized value of iHS was calculated in each group and plotted as (A) Group I, (B) Group II-A, and (C) Group II-B.
(TIF)

Figure S12 **Chromosome ideograms of genome-wide integrated haplotype homozygosity (|iHS|).** Red bar shows iHS in Group I. Blue and orange bar indicate iHS in Groups II-A and II-B, respectively. Only |iHS| > 2.7 is shown.
(TIF)

Figure S13 **Genome-wide plot of |iHS| of Group I.**
(TIF)
