# Supplementary material for: Effect of Artificial Selection on Runs of Homozygosity in U.S. Holstein Cattle
Source: PLoS One. 2013 Nov 14;8(11):e80813. doi: 10.1371/journal.pone.0080813 (PMC3858116; doi:10.1371/journal.pone.0080813)
Supplement: Table S1 — Summary statistics of FP and FG. (DOCX) [file pone.0080813.s001.docx]

**Table S1. Summary statistics of *F_P_* and *F_G_*.**

|  | Group I | | Group II-A | | Group II-B | |
| --- | --- | --- | --- | --- | --- | --- |
|  | *F_P_*^1^ | *F_G_*^2^ | *F_P_*^1^ | *F_G_*^2^ | *F_P_*^1^ | *F_G_*^2^ |
| Mean | 0.016^*^ | 0.058^**^ | 0.045 | 0.086 | 0.043 | 0.079 |
| Median | 0.011 | 0.051 | 0.046 | 0.084 | 0.042 | 0.076 |
| s.d.^3^ | 0.017 | 0.03 | 0.023 | 0.03 | 0.014 | 0.028 |
| Maximum | 0.08 | 0.198 | 0.299 | 0.293 | 0.081 | 0.191 |

^1^Includes founder animals (*F_P_*=0)

^2^*F_G_* at 50 SNP ROH

^3^standard deviation

*significant differences from Group II (p < 0.001)

**significant differences from Group II (p < 0.0001)
