# Supplementary material for: Effect of Artificial Selection on Runs of Homozygosity in U.S. Holstein Cattle
Source: PLoS One. 2013 Nov 14;8(11):e80813. doi: 10.1371/journal.pone.0080813 (PMC3858116; doi:10.1371/journal.pone.0080813)
Supplement: Table S2 — Correlations (r) between FP and FG. (DOCX) [file pone.0080813.s002.docx]

**Table S2. Correlations (*r*) between *F_P_* and *F_G_***

| **Threshold** | **Group I^*^** | **Group II-A** | **Group II-B** | **All Groups^*^** |
| --- | --- | --- | --- | --- |
| 50 SNP | 0.62 | 0.68 | 0.59 | 0.64 |
| 100 SNP | 0.60 | 0.64 | 0.58 | 0.61 |

^*^Estimation excluding the 100 animals with *F_p_* = 0 in Group I
