# Supplementary material for: Effect of Artificial Selection on Runs of Homozygosity in U.S. Holstein Cattle
Source: PLoS One. 2013 Nov 14;8(11):e80813. doi: 10.1371/journal.pone.0080813 (PMC3858116; doi:10.1371/journal.pone.0080813)
Supplement: Table S3 — Linear regression of FG on FP . (DOCX) [file pone.0080813.s003.docx]

**Table S3. Linear regression of *F_G_* on *F_P_* .**

|  | **Group I** | | **Group II-A** | | **Group II-B** | |
| --- | --- | --- | --- | --- | --- | --- |
|  | 50 SNP | 100 SNP | 50 SNP | 100 SNP | 50 SNP | 100 SNP |
| *F_P_* of founder^1^ | 0.037 | 0.021 | 0.042 | 0.025 | 0.035 | 0.02 |
| (s.d.) | 0.004 | 0.003 | 0.002 | 0.002 | 0.005 | 0.005 |
| Slope^2^ | 1.04 | 1.005 | 0.898 | 0.851 | 1.02 | 0.951 |
| (s.d.) | 0.116 | 0.106 | 0.03 | 0.029 | 0.116 | 0.115 |

^1^Intercept of regression

^2^Slope of regression of *F_G_* on *F_P_*
