# Supplementary material for: Effect of Artificial Selection on Runs of Homozygosity in U.S. Holstein Cattle
Source: PLoS One. 2013 Nov 14;8(11):e80813. doi: 10.1371/journal.pone.0080813 (PMC3858116; doi:10.1371/journal.pone.0080813)
Supplement: Table S4 — Mean locus homozygosity (FL). (DOCX) [file pone.0080813.s004.docx]

**Table S4. Mean locus homozygosity (*F_L_*).**

| **Chr** | **Group I^*^** | **(s.d.)** | **Group II-A** | **(s.d.)** | **Group II-B** | **(s.d.)** |
| --- | --- | --- | --- | --- | --- | --- |
| 1 | 0.063 | 0.03 | 0.093 | 0.03 | 0.103 | 0.04 |
| 2 | 0.052 | 0.02 | 0.094 | 0.03 | 0.098 | 0.03 |
| 3 | 0.062 | 0.03 | 0.076 | 0.02 | 0.076 | 0.04 |
| 4 | 0.05 | 0.02 | 0.072 | 0.03 | 0.072 | 0.03 |
| 5 | 0.037 | 0.01 | 0.068 | 0.03 | 0.074 | 0.04 |
| 6 | 0.065 | 0.02 | 0.083 | 0.03 | 0.084 | 0.05 |
| 7 | 0.067 | 0.03 | 0.104 | 0.03 | 0.093 | 0.03 |
| 8 | 0.048 | 0.02 | 0.093 | 0.03 | 0.085 | 0.04 |
| 9 | 0.035 | 0.02 | 0.073 | 0.02 | 0.078 | 0.03 |
| 10 | 0.058 | 0.02 | 0.113 | 0.04 | 0.078 | 0.04 |
| 11 | 0.052 | 0.02 | 0.075 | 0.02 | 0.063 | 0.02 |
| 12 | 0.051 | 0.02 | 0.072 | 0.02 | 0.055 | 0.02 |
| 13 | 0.082 | 0.04 | 0.112 | 0.04 | 0.101 | 0.06 |
| 14 | 0.071 | 0.03 | 0.086 | 0.02 | 0.078 | 0.03 |
| 15 | 0.043 | 0.02 | 0.055 | 0.01 | 0.054 | 0.03 |
| 16 | 0.072 | 0.03 | 0.101 | 0.03 | 0.096 | 0.04 |
| 17 | 0.051 | 0.02 | 0.073 | 0.02 | 0.076 | 0.03 |
| 18 | 0.054 | 0.02 | 0.047 | 0.01 | 0.038 | 0.02 |
| 19 | 0.051 | 0.02 | 0.067 | 0.02 | 0.065 | 0.02 |
| 20 | 0.068 | 0.02 | 0.125 | 0.06 | 0.098 | 0.04 |
| 21 | 0.047 | 0.02 | 0.073 | 0.02 | 0.064 | 0.02 |
| 22 | 0.052 | 0.03 | 0.08 | 0.03 | 0.098 | 0.06 |
| 23 | 0.057 | 0.02 | 0.077 | 0.03 | 0.089 | 0.03 |
| 24 | 0.062 | 0.03 | 0.084 | 0.03 | 0.074 | 0.04 |
| 25 | 0.043 | 0.01 | 0.066 | 0.01 | 0.049 | 0.02 |
| 26 | 0.061 | 0.03 | 0.09 | 0.05 | 0.124 | 0.05 |
| 27 | 0.046 | 0.01 | 0.054 | 0.01 | 0.058 | 0.01 |
| 28 | 0.044 | 0.01 | 0.059 | 0.02 | 0.069 | 0.02 |
| 29 | 0.044 | 0.02 | 0.076 | 0.03 | 0.05 | 0.03 |

^*^Mean *F_L_* of each chromosome at ROH threshold of 50 SNP = sum of *F_L_*/number of SNP loci of the chromosome
