# Supplementary material for: Effect of Artificial Selection on Runs of Homozygosity in U.S. Holstein Cattle
Source: PLoS One. 2013 Nov 14;8(11):e80813. doi: 10.1371/journal.pone.0080813 (PMC3858116; doi:10.1371/journal.pone.0080813)
Supplement: Table S5 — Genomic regions reaching top 3% of all FL across groups. (DOCX) [file pone.0080813.s005.docx]

**Table S5. Genomic regions reaching top 3% of all *F_L_* across groups.**

| **Chr** | **Group I (Mb)** |  | **Group II-A (Mb)^*^** |  | **Group II-B (Mb)** |
| --- | --- | --- | --- | --- | --- |
| 1 | - |  | - |  | 16.25-18.23 |
|  | - |  | 29.70-32.78 |  | 27.79-32.78 |
|  | - |  | 48.13-53.53 |  | 47.70-53.53 |
|  | - |  | - |  | 81.89-85.70 |
| 2 | - |  | 129.16-133.03 |  | 128.70-133.16 |
| 3 | - |  | 69.90-72.22 |  | 69.90-72.22 |
| 5 | - |  | 97.22-97.38 |  | 98.34-101.40 |
| 6 | - |  | 75.30-76.13 |  | 73.28-89.13 |
| 7 | - |  | 42.44-46.73 |  | 42.87-45.87 |
| 8 | - |  | - |  | 94.41-96.32 |
| 10 | - |  | 36.11-38.75 |  | 38.52-38.75 |
|  | - |  | 49.75-61.06 |  | - |
|  | - |  | 70.11-72.06 |  | 70.11-72.23 |
| 11 | - |  | 6.32-7.46 |  | - |
| 13 | 36.58-37.24 |  | 29.04-34.98 |  | - |
|  | 46.91-50.66 |  | 46.15-49.62 |  | 45.85-51.17 |
|  | - |  | 54.04-56.75 |  | 53.31-57.32 |
| 14 | - |  | - |  | 18.88-21.58 |
|  | - |  | - |  | 27.81-28.58 |
| 16 | - |  | 58.58-60.37 |  | - |
|  | - |  | - |  | 66.22-68.75 |
|  | - |  | - |  | 69.50-72.14 |
| 20 | - |  | 24.06-49.96 |  | 24.67-25.64 |
|  | - |  | - |  | 36.56-36.92 |
| 22 | - |  | 22.38-22.56 |  | 14.25-26.16 |
| 23 | - |  | 28.90-31.46 |  | - |
| 24 | - |  | - |  | 41.07-45.7 |
| 26 | 21.22-21.87 |  | 20.01-24.03 |  | 17.28-24.58 |
|  | - |  | - |  | 35.62-41.35 |
| 29 | - |  | 42.62-43.38 |  | 41.85-43.84 |

^*^ Top 1% = 0.193; 2%=0.163; 3%=0.155; 4%=0.144; 5%=0.139
