# Supplementary material for: Effect of Artificial Selection on Runs of Homozygosity in U.S. Holstein Cattle
Source: PLoS One. 2013 Nov 14;8(11):e80813. doi: 10.1371/journal.pone.0080813 (PMC3858116; doi:10.1371/journal.pone.0080813)
Supplement: Table S6 — Genomic intervals representing differences in FL between groups*. (DOCX) [file pone.0080813.s006.docx]

**Table S6. Genomic intervals representing differences in *F_L_* between groups^*^.**

| **BTA** | **Group II-A vs I** |  | **Group II-B vs I** |  | **Group II-A vs II-B** |
| --- | --- | --- | --- | --- | --- |
| 1 | 50.68-53.53 |  | 50.68-52.80 |  | - |
|  | 77.55-80.47 |  | 77.55-80.14 |  | - |
| 2 | 117.95-118.28 |  | 117.95-117.98 |  | - |
|  | 123.56-123.77 |  | - |  | - |
|  | 129.16-135.59 |  | 129.49-135.59 |  | - |
| 4 | 113.74-113.94 |  | - |  | - |
|  | 118.16-118.56 |  | - |  | - |
| 5 | - |  | - |  | 114.82-116.55 |
| 6 | - |  | - |  | 4.31-7.15 |
| 8 | - |  | - |  | 43.24-44.76 |
|  | 46.41-46.99 |  | - |  | - |
| 9 | 57.71-57.94 |  | 57.71-57.94 |  | - |
|  | - |  | 59.65-61.24 |  | - |
|  | 67.72-76.15 |  | 67.72-75.08 |  | - |
|  | 91.86-92.49 |  | - |  | - |
| 11 | - |  | 30.16-79.85^**^ |  | 30.16-79.85 |
|  | - |  | - |  | - |
| 12 | - |  | - |  | 21.18-21.89 |
|  | - |  | - |  | 22.88-23.35 |
| 14 | - |  | - |  | 82.74-84.07 |
| 15 | - |  | - |  | 12.08-12.82 |
| 18 | - |  | 3.96-4.46 |  | 3.78-4.46 |
|  | - |  | 25.19-28.03^**^ |  | 25.19-27.28 |
|  | - |  | 38.62-39.20^**^ |  | 38.62-39.20 |
| 21 | 26.93-27.92 |  | 26.93-27.92 |  | - |
| 22 | 7.38-8.69 |  | 7.38-8.69 |  | - |
| 24 | - |  | 9.76-10.34^**^ |  | 8.39-10.56 |
| 26 | - |  | 36.06-36.10 |  |  |
|  | - |  | 41.64-41.82 |  | - |
|  | - |  | 47.96-48.30^**^ |  | 47.96-48.30 |
| 29 |  |  | 28.88-30.06^**^ |  | 27.65-30.98 |

^*^Regions with an absolute standardized score sln(*F_L_*) > 3.0

^**^Raw score is negative
