# Supplementary material for: Effect of Artificial Selection on Runs of Homozygosity in U.S. Holstein Cattle
Source: PLoS One. 2013 Nov 14;8(11):e80813. doi: 10.1371/journal.pone.0080813 (PMC3858116; doi:10.1371/journal.pone.0080813)
Supplement: Table S7 — Summary of genome-wide |iHS|. (DOCX) [file pone.0080813.s007.docx]

**Table S7. Summary of genome-wide |iHS|.**

| **Group** | **Chr** | **Candidate region** | | **Number of SNP** | **Max \|iHS\|** | **Pos (max)** |
| --- | --- | --- | --- | --- | --- | --- |
| Group I | 3 | 60.63 | 63.02 | 16 | 2.64 | 63.02 |
|  |  | 68.26 | 69.68 | 16 | 3.21 | 69.11 |
|  |  | 75.72 | 77.11 | 12 | 2.91 | 76.71 |
|  | 7 | 41.32 | 45.14 | 30 | 3.13 | 44.59 |
|  |  | 72.05 | 77.63 | 55 | 3.81 | 74.89 |
|  | 8 | 79.90 | 81.77 | 11 | 2.75 | 78.61 |
|  | 13 | 36.23 | 41.01 | 37 | 3.13 | 38.68 |
|  |  | 42.00 | 44.26 | 19 | 3.66 | 42.00 |
|  |  | 45.39 | 49.62 | 42 | 3.53 | 45.99 |
|  |  | 54.37 | 57.23 | 22 | 2.99 | 55.41 |
|  |  | 57.84 | 67.82 | 70 | 3.99 | 63.01 |
|  | 14 | 26.91 | 29.04 | 20 | 2.81 | 28.44 |
|  | 17 | 2.48 | 10.09 | 64 | 3.65 | 4.38 |
|  | 19 | 53.58 | 55.06 | 12 | 2.94 | 55.04 |
|  | 22 | 19.68 | 22.59 | 23 | 3.42 | 21.67 |
|  |  | 23.33 | 25.34 | 15 | 2.86 | 25.34 |
|  |  | 55.94 | 57.94 | 17 | 2.65 | 56.97 |
|  | 26 | 23.46 | 25.58 | 11 | 2.99 | 24.29 |
|  | 29 | 16.45 | 18.00 | 17 | 2.97 | 18.18 |
| Group II-A | 1 | 29.29 | 32.63 | 19 | 3.09 | 29.36 |
|  |  | 49.71 | 52.44 | 18 | 3.18 | 51.09 |
|  | 2 | 113.95 | 115.66 | 11 | 2.91 | 114.96 |
|  |  | 121.83 | 125.11 | 20 | 2.86 | 91.25 |
|  |  | 129.87 | 132.86 | 24 | 3.5 | 131.92 |
|  |  | 133.87 | 135.86 | 19 | 3.28 | 135.48 |
|  | 6 | 9.08 | 10.91 | 14 | 3.3 | 9.3 |
|  | 7 | 40.83 | 43.30 | 25 | 3.3 | 41.32 |
|  |  | 95.42 | 97.12 | 11 | 3.2 | 96.36 |
|  | 9 | 88.08 | 89.55 | 10 | 2.95 | 89 |
|  | 10 | 12.9 | 13.97 | 10 | 2.9 | 13.97 |
|  |  | 48.9 | 51.52 | 28 | 3.86 | 49.5 |
|  |  | 52.79 | 55.11 | 15 | 2.73 | 52.92 |
|  |  | 58.16 | 60.34 | 12 | 2.83 | 59.98 |
|  |  | 68.36 | 70.55 | 14 | 2.9 | 70.55 |
|  | 11 | 5.18 | 8.35 | 18 | 3.05 | 6.62 |
|  | 14 | 9.86 | 11.20 | 10 | 2.83 | 10.49 |
|  | 20 | 24.51 | 28.41 | 21 | 3.17 | 27.01 |
|  | 21 | 6.91 | 7.95 | 12 | 3.64 | 73.5 |
|  | 24 | 31.53 | 33.40 | 13 | 3.23 | 33.11 |
| Group II-B | 1 | 49.55 | 52.75 | 14 | 2.83 | 51.51 |
|  | 2 | 127.9 | 132.2 | 27 | 3.3 | 130.00 |
|  | 3 | 70.25 | 73.89 | 19 | 3.35 | 71.70 |
|  | 5 | 97.09 | 105.95 | 32 | 2.96 | 101.69 |
|  | 7 | 91.03 | 99.05 | 37 | 3.29 | 95.90 |
|  | 8 | 93.04 | 97.44 | 29 | 2.82 | 94.99 |
|  | 10 | 49.34 | 51.59 | 19 | 2.82 | 50.74 |
|  | 13 | 46.2 | 50.69 | 35 | 3.42 | 48.43 |
|  |  | 54.37 | 56.37 | 15 | 3.07 | 55.37 |
|  | 16 | 60.42 | 64.74 | 22 | 2.59 | 62.05 |
|  | 20 | 24.3 | 27.47 | 14 | 2.97 | 25.83 |
|  | 24 | 31.53 | 40.96 | 53 | 3.34 | 35.71 |
|  | 26 | 38.92 | 41.81 | 17 | 3.12 | 38.98 |
