# Supplementary figures and images for: Effect of Artificial Selection on Runs of Homozygosity in U.S. Holstein Cattle
Source: PLoS One. 2013 Nov 14;8(11):e80813. doi: 10.1371/journal.pone.0080813 (PMC3858116; doi:10.1371/journal.pone.0080813)

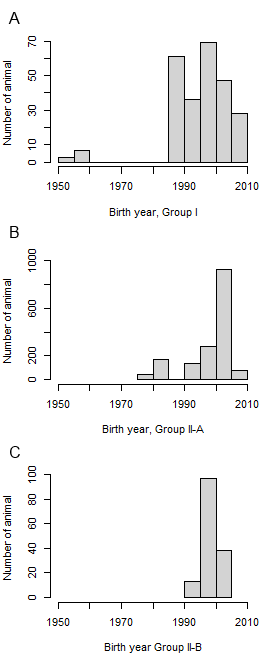

Supplement: Figure S1 — Distribution of Birth Year across groups. The total animal counts (y-axis) is plotted against birthyear (x-axis) for each group. (TIF) [file pone.0080813.s008.tif]

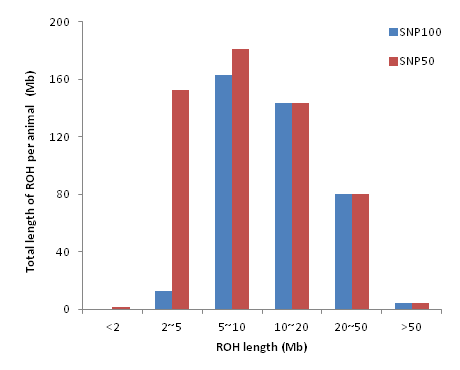

Supplement: Figure S2 — Distribution of ROH size across groups. The total counts of ROH detected across groups with ROH sizes binned on the x-axis and counts indicated on the y-axis using a 50 (red) or 100 (blue) SNP threshold. (TIF) [file pone.0080813.s009.tif]

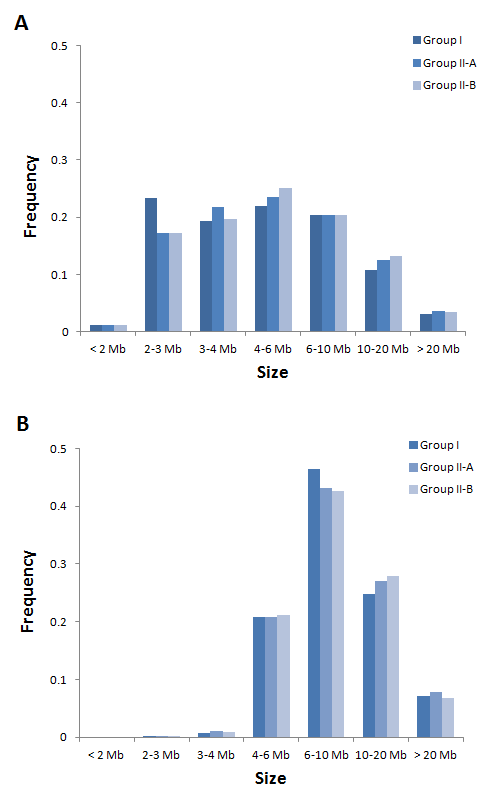

Supplement: Figure S3 — Frequency of ROH sizes detected by group. ROH lengths were binned by size within group by threshold of a A) 50 SNP or B) 100 SNP window. The y-axis represents frequency of ROH length detected as a percentage of all ROH detected within a group. Length bins are grouped on the x-axis. (TIF) [file pone.0080813.s010.tif]

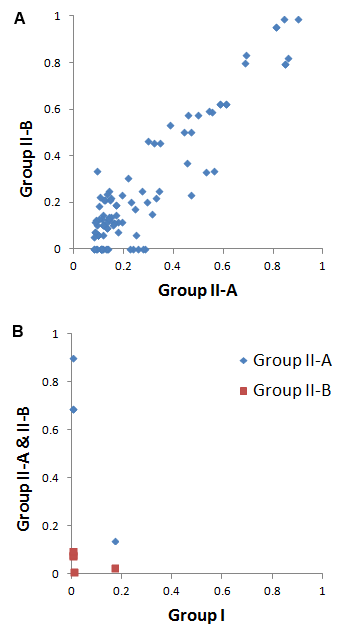

Supplement: Figure S4 — Correlation of common pedigree ancestors between groups. The appearance of genotyped descendants from common influential sires were counted and expressed as a percentage of the entire group. The graphs comparing the percentage influence from common sires includes: A) common sires from Groups II-A (y-axis) versus II-B (x-axis) and B) Group II (y-axis) versus Group I (x-axis). (TIF) [file pone.0080813.s011.tif]

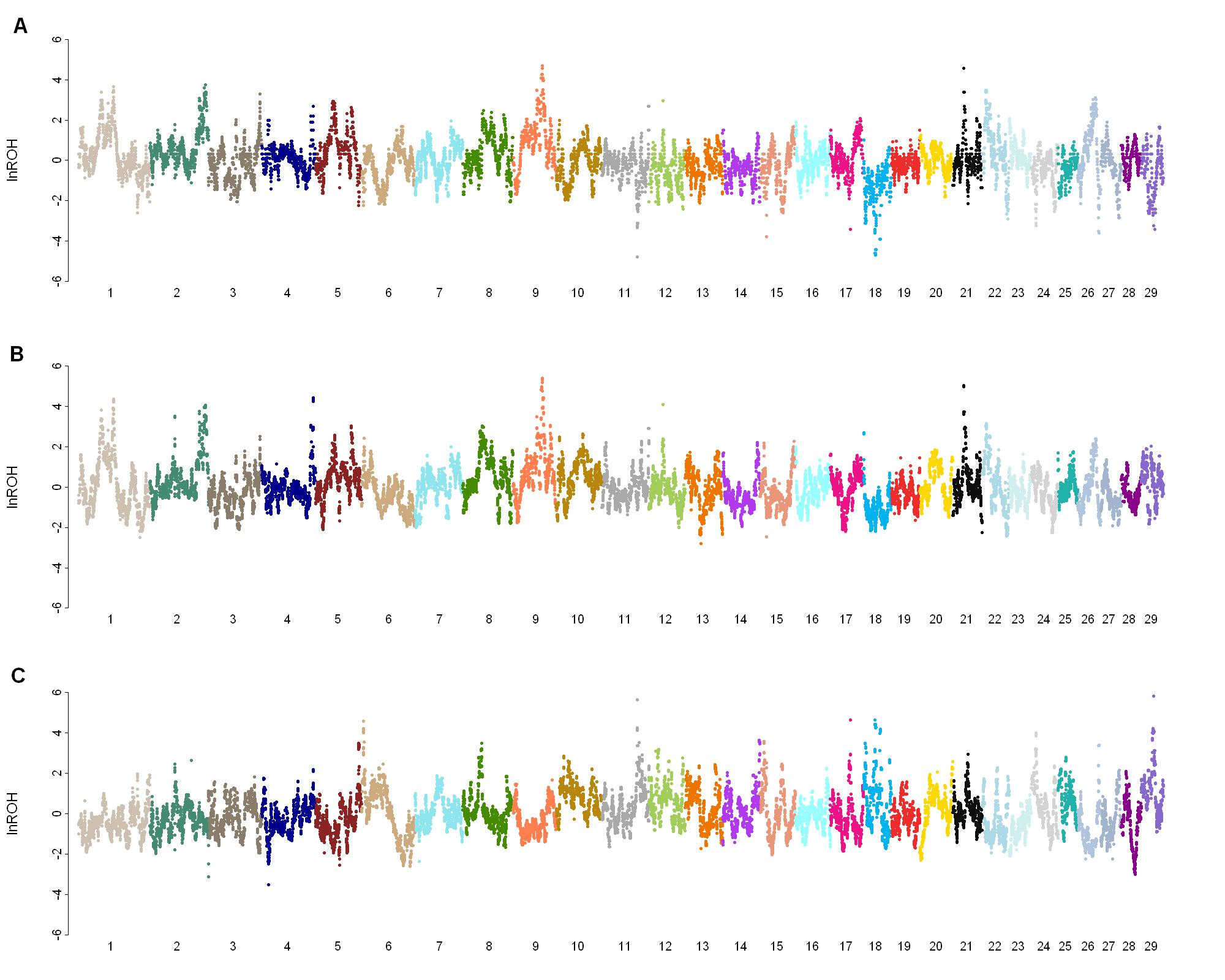

Supplement: Figure S5 — Genome wide plot of comparative FL. Manhattan plots were generated using the standardized log ratio of F L values (y-axis) for each SNP locus relative to its genome coordinate (x-axis; Chr indicated) for A) Group II-A vs I, B) Group II-B vs I, and C) Group II-A vs II-B. (TIF) [file pone.0080813.s012.tif]

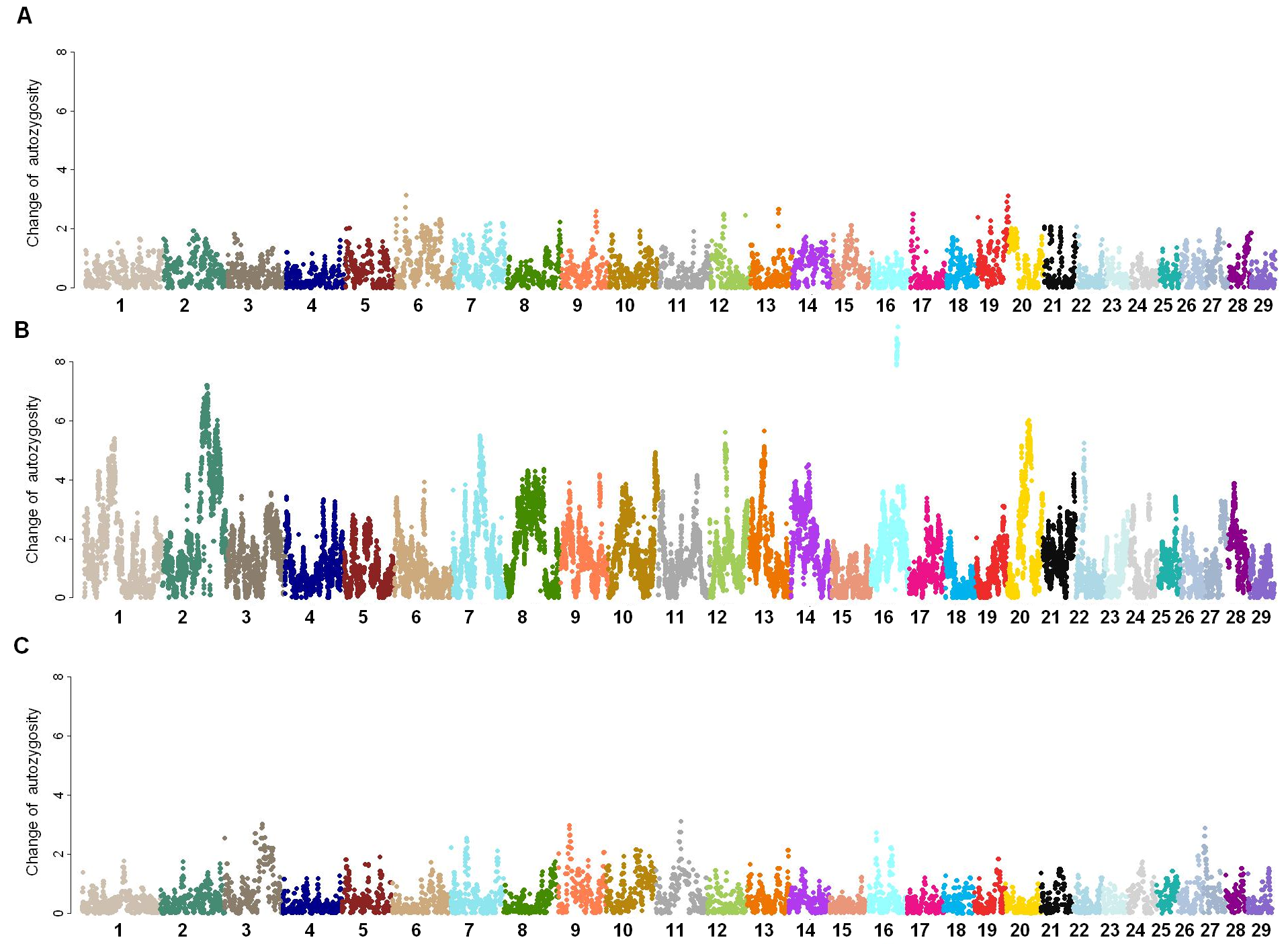

Supplement: Figure S6 — Genome-wide changes of autozygosity within Groups. Manhattan plots of change in autozygosity were generated using (ΔFL) for each SNP locus (y-axis) relative to its genome coordinate (x-axis; Chr indicated) for A) Group I (1953~2006), B) Group II-A (1975~2007), and C) Group II-B (1987~2003). (TIF) [file pone.0080813.s013.tif]

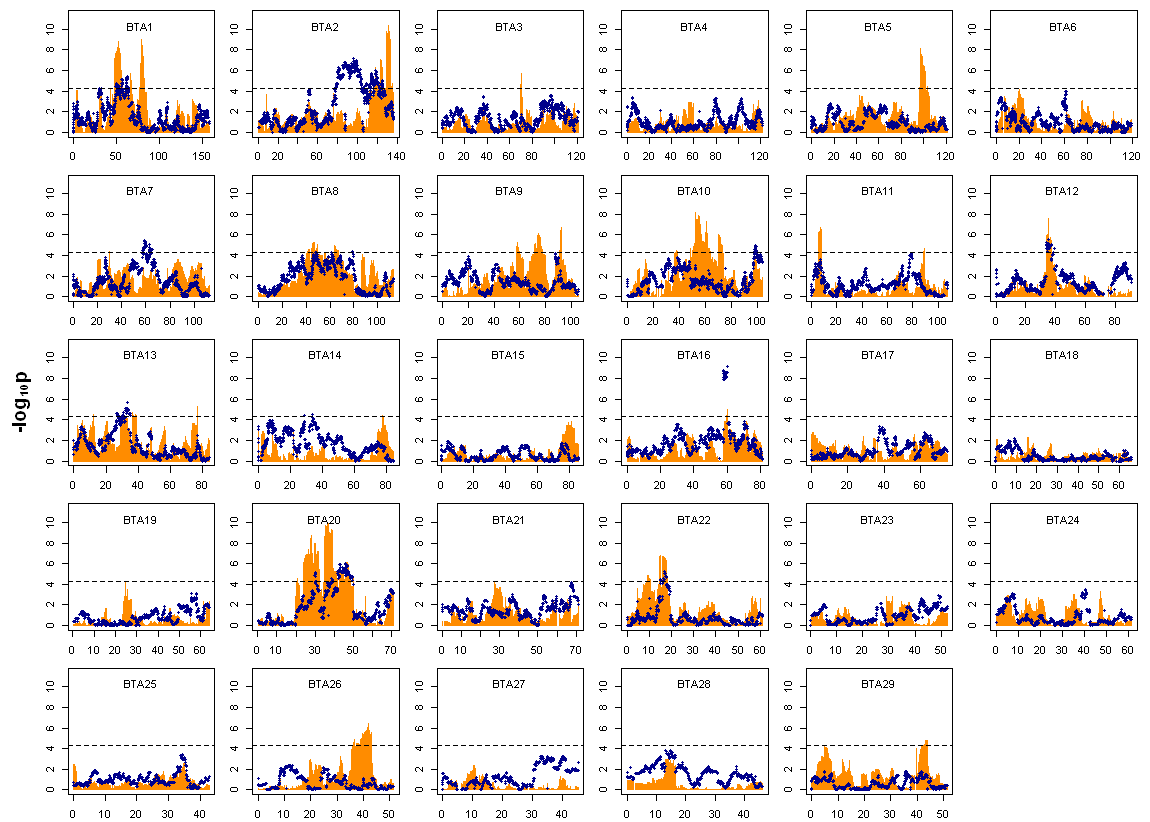

Supplement: Figure S7 — Comparison of change of autozygosity and differential selection of autozygosity. Genome-wide plots by chromosome of–log 10 p values (y-axis) of the change of autozygosity (ΔF L - blue dots) of Group II-A and differential selection on autozygosity (orange bars) between Groups II-A and I. (TIF) [file pone.0080813.s014.tif]

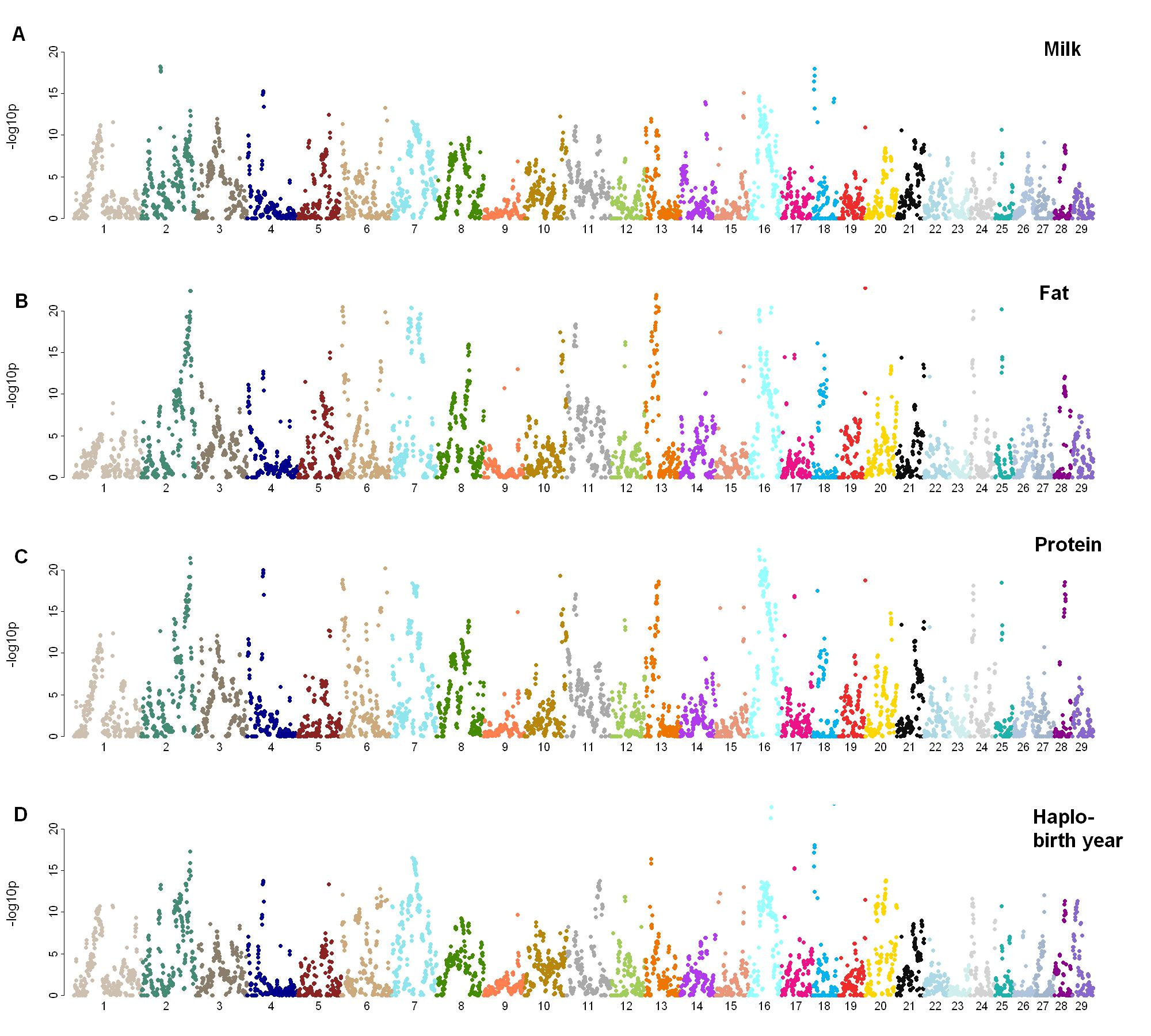

Supplement: Figure S8 — Comparison between change of the most frequent haplotype and haplotype-trait associations in Group II-A. Genome-wide Manhattan plots by chromosome of–log 10 p values (y-axis) of the association between the most frequent haaplotype and haplotype trait associations. Plots are for the following traits A) Milk, B) Fat, and C) Protein, while D) plots haplotype by birth year. (TIF) [file pone.0080813.s015.tif]

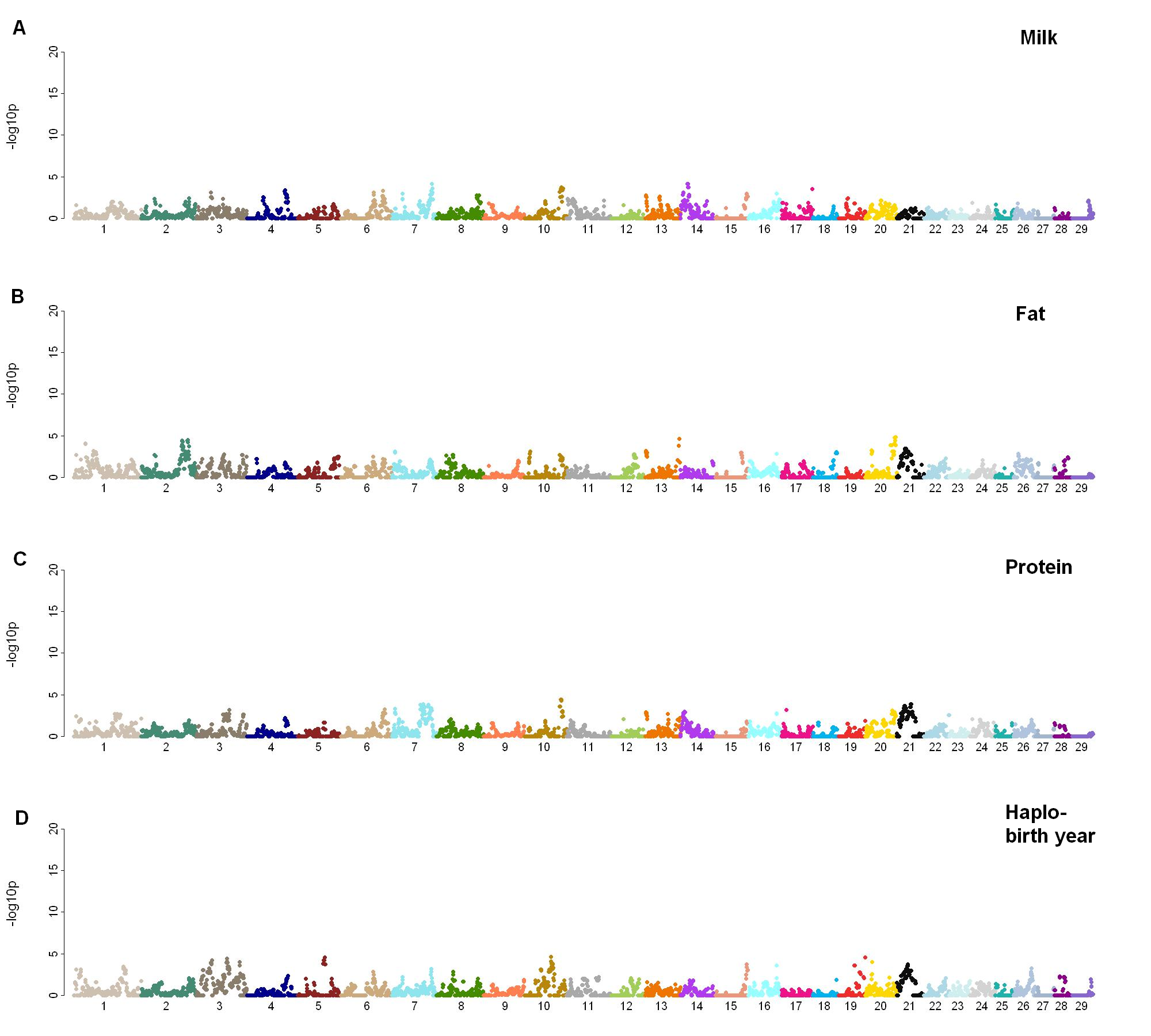

Supplement: Figure S9 — Comparison between change of the most frequent haplotype and haplotype-trait associations in Group II-B. Genome-wide Manhattan plots by chromosome of–log 10 p values (y-axis) of the association between the most frequent haaplotype and haplotype trait associations. Plots are for the following traits A) Milk, B) Fat, and C) Protein, while D) plots haplotype by birth year. (TIF) [file pone.0080813.s016.tif]

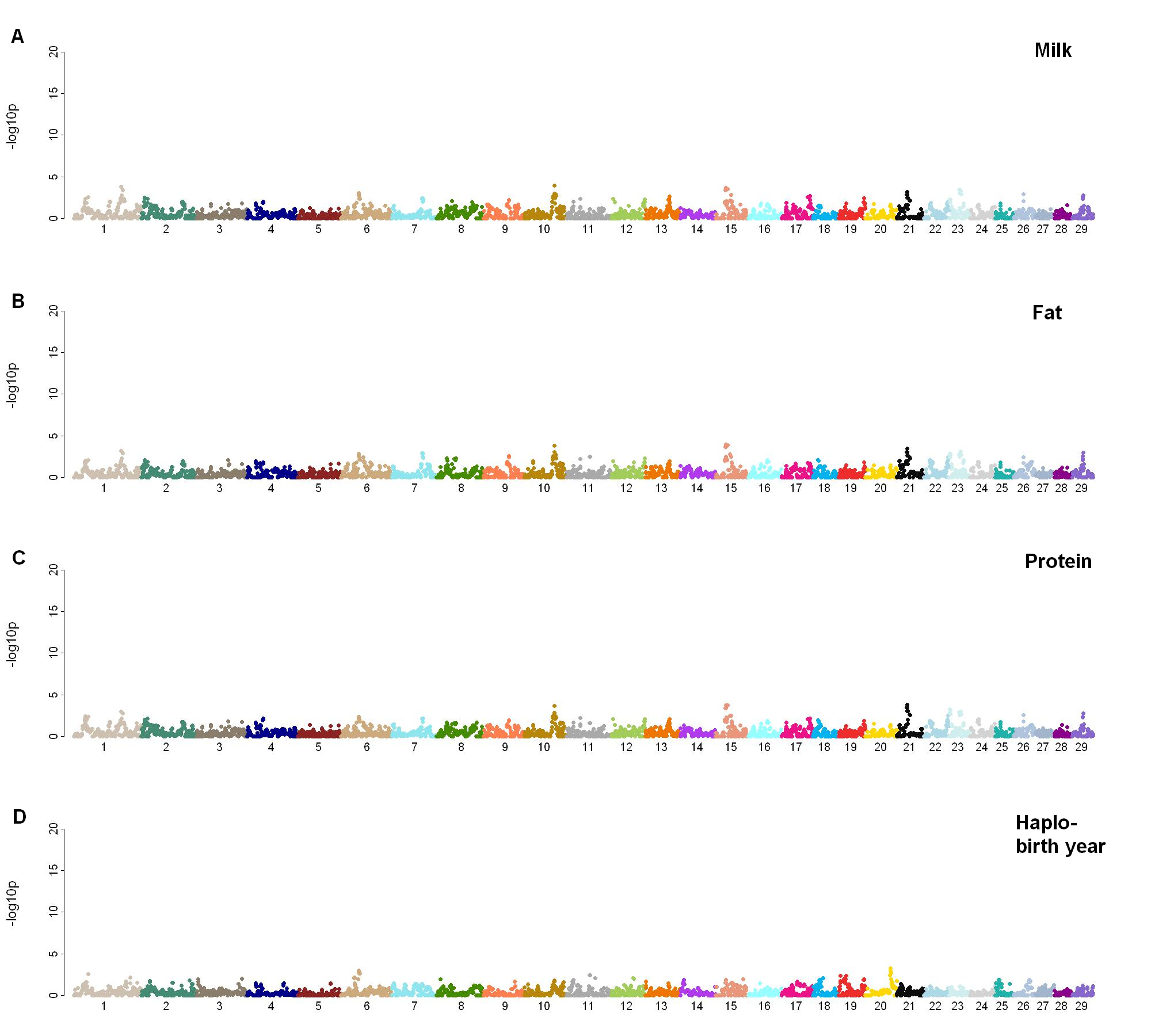

Supplement: Figure S10 — Comparison between change of the most frequent haplotype and haplotype-trait associations in Group I. Genome-wide Manhattan plots by chromosome of–log 10 p values (y-axis) of the association between the most frequent haaplotype and haplotype trait associations. Plots are for the following traits A) Milk, B) Fat, and C) Protein, while D) plots haplotype by birth year. (TIF) [file pone.0080813.s017.tif]

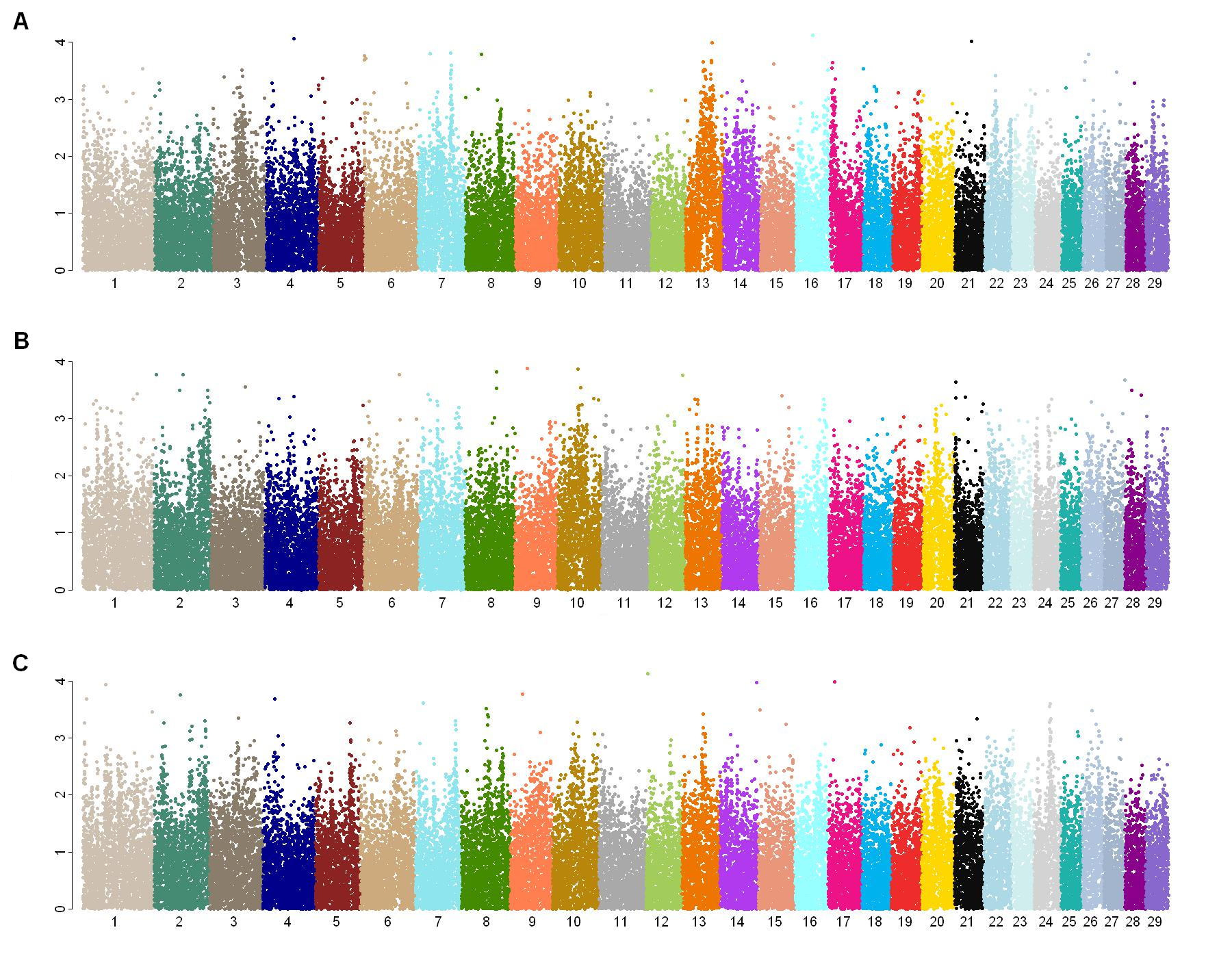

Supplement: Figure S11 — Genome-wide integrated extended haplotype homozygosity. Absolute values of iHS were plotted across the genome. The standardized value of iHS was calculated in each group and plotted as (A) Group I, (B) Group II-A, and (C) Group II-B. (TIF) [file pone.0080813.s018.tif]

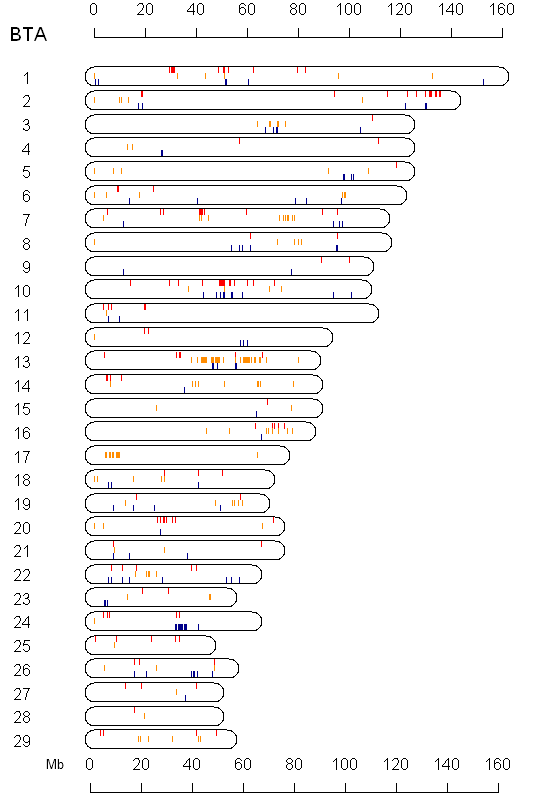

Supplement: Figure S12 — Chromosome ideograms of genome-wide integrated haplotype homozygosity (|iHS|). Red bar shows iHS in Group I. Blue and orange bar indicate iHS in Groups II-A and II-B, respectively. Only |iHS| > 2.7 is shown. (TIF) [file pone.0080813.s019.tif]

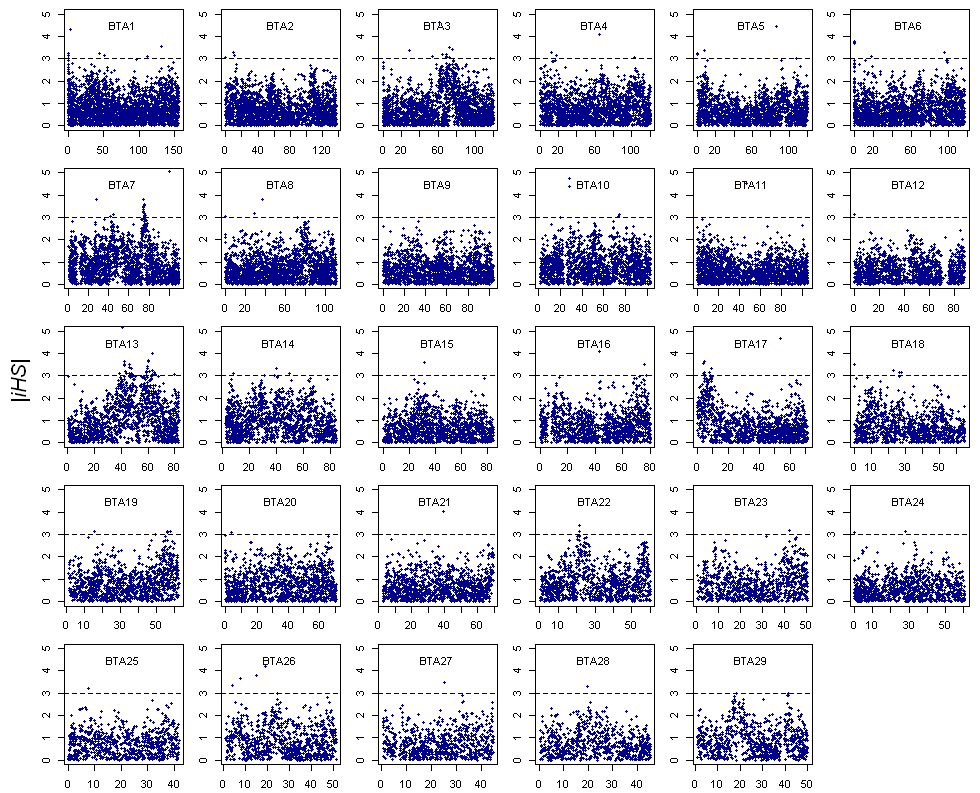

Supplement: Figure S13 — Genome-wide plot of |iHS| of Group I. (TIF) [file pone.0080813.s020.tif]
